# Inversion of a topological domain leads to restricted changes in its gene expression and affects interdomain communication

**DOI:** 10.1242/dev.200568

**Published:** 2022-05-06

**Authors:** Rafael Galupa, Christel Picard, Nicolas Servant, Elphège P. Nora, Yinxiu Zhan, Joke G. van Bemmel, Fatima El Marjou, Colin Johanneau, Maud Borensztein, Katia Ancelin, Luca Giorgetti, Edith Heard

**Affiliations:** 1 Mammalian Developmental Epigenetics Group, Genetics and Developmental Biology Unit, Institut Curie, PSL Research University, CNRS UMR3215, INSERM U934, Paris 75005, France; 2 Bioinformatics, Biostatistics, Epidemiology and Computational Systems Unit, Institut Curie, PSL Research University, INSERM U900, Paris 75005, France; 3 MINES ParisTech, PSL Research University, CBIO-Centre for Computational Biology, Paris 75006, France; 4 Friedrich Miescher Institute for Biomedical Research, Basel 4058, Switzerland; 5 University of Basel, Basel 4001, Switzerland; 6 Transgenesis Facility, Institut Curie, Paris 75005, France; 7 Collège de France, Paris 75231, France

**Keywords:** TADs, Gene expression, X-inactivation, Xist, Genomic engineering

## Abstract

The interplay between the topological organization of the genome and the regulation of gene expression remains unclear. Depletion of molecular factors (e.g. CTCF) underlying topologically associating domains (TADs) leads to modest alterations in gene expression, whereas genomic rearrangements involving TAD boundaries disrupt normal gene expression and can lead to pathological phenotypes. Here, we targeted the TAD neighboring that of the noncoding transcript *Xist*, which controls X-chromosome inactivation. Inverting 245 kb within the TAD led to expected rearrangement of CTCF-based contacts but revealed heterogeneity in the ‘contact’ potential of different CTCF sites. Expression of most genes therein remained unaffected in mouse embryonic stem cells and during differentiation. Interestingly, expression of *Xist* was ectopically upregulated. The same inversion in mouse embryos led to biased *Xist* expression. Smaller inversions and deletions of CTCF clusters led to similar results: rearrangement of contacts and limited changes in local gene expression, but significant changes in *Xist* expression in embryos. Our study suggests that the wiring of regulatory interactions within a TAD can influence the expression of genes in neighboring TADs, highlighting the existence of mechanisms of inter-TAD communication.

## Abstract (precis)

**Summary:** Using genomic engineering in mouse embryos and embryonic stem cells, expression of *Xist*, which regulates X-inactivation, is found to be sensitive to topological changes in the neighboring TAD.

## INTRODUCTION

The three-dimensional folding of the genome has been increasingly recognized as an essential component for our understanding of gene regulation (Dekker and Mirny, 2016; McCord et al., 2020). Chromosome conformation capture techniques (Denker and de Laat, 2016) have unraveled a complex hierarchy of structural layers that organize mammalian chromosomes, composed of domains of high-frequency contacts (Zhan et al., 2017). At the sub-megabase level, these domains are generally designated topologically associating domains (TADs) (Dixon et al., 2012; Nora et al., 2012) and are well conserved across species and invariant across cell types (Dekker and Heard, 2015). The dynamics of the formation and maintenance of TADs and their boundaries during development and each cell cycle remains under investigation (Szabo et al., 2019) but appears to depend on the interplay between the architectural proteins cohesin and the zinc finger protein CTCF (Fudenberg et al., 2016; Haarhuis et al., 2017; Nora et al., 2017; Rao et al., 2017; Sanborn et al., 2015; Schwarzer et al., 2017; Wutz et al., 2017). Enriched at boundaries between TADs (Dixon et al., 2012; Phillips-Cremins et al., 2013), CTCF is required for chromatin loops observed between CTCF sites and for the organization and insulation of most TADs (Nora et al., 2017). Remarkably, CTCF-mediated contacts mainly occur between CTCF sites in which the CTCF motifs lie in a convergent orientation (Rao et al., 2014; Tang et al., 2015), and the contacts depend on the orientation of the motifs: altering the orientation of a CTCF site can disrupt a loop and lead to the formation of new ones (de Wit et al., 2015; Guo et al., 2015; Sanborn et al., 2015).

TADs are thought to instruct gene regulatory landscapes, allowing promoters and their regulatory elements to meet often and lead to a more efficient transcriptional output (Symmons et al., 2016). Accordingly, TADs represent the folding scale at which promoter-enhancer interactions and gene co-regulation are maximized (Zhan et al., 2017). The communication between promoters and enhancers is generally assumed to rely on chromatin looping, and long-range contacts within TADs can be dynamic during processes that involve rewiring of the regulatory networks, such as differentiation (Dixon et al., 2015). However, the interplay between such topological organization and the regulation of gene expression remains unclear. Loss of TADs upon depletion of CTCF or cohesin leads to relatively small effects on gene expression (Nora et al., 2017; Rao et al., 2017; Schwarzer et al., 2017; Wutz et al., 2017), and genomic rearrangements involving mammalian TADs and their boundaries can have either very modest effects (Amândio et al., 2020; Despang et al., 2019; Paliou et al., 2019; Rodríguez-Carballo et al., 2017; Williamson et al., 2019) or disrupt normal gene expression and underlie pathological phenotypes (Flavahan et al., 2016; Franke et al., 2016; Hnisz et al., 2016; Lupiáñez et al., 2015).

**Fig. 1.**
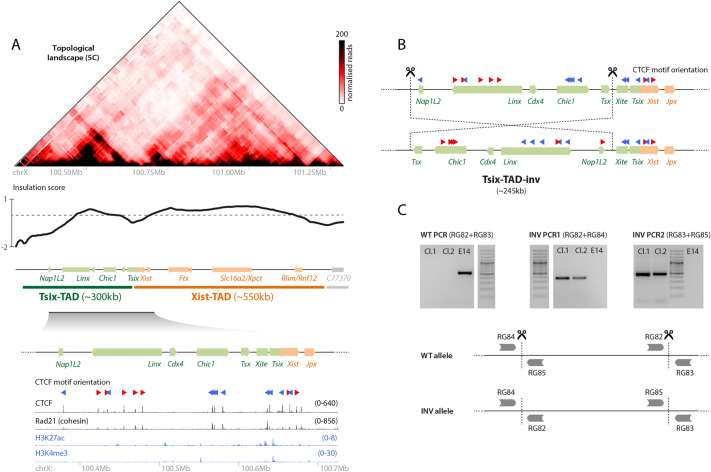
**Strategy for inverting almost the entire Tsix-TAD.** (A) Topological organization of the *Xic* (top) and chromatin ChIP-seq profiles (bottom; see Materials and Methods for sources); the *Xist*/*Tsix* locus lies at the boundary between two TADs. The red and blue arrowheads indicate the orientation of the CTCF motif (orientated left or right, respectively). (B) Targeting strategy for inverting the ∼245 kb region comprising most of the Tsix-TAD, except *Tsix* and its known regulator *Xite*, and leaving the boundaries intact. (C) PCR strategy (bottom) and gel results (top) for detecting the inversion events. E14 is the wild-type (WT) parental cell line. Cl.1 and Cl.2 are the two clones that were generated and analyzed throughout the study.

Here, we explored the relationship between TAD organization and transcriptional regulation in a crucial developmental regulatory landscape, the mouse X-inactivation center (*Xic*). The *Xic* is the master regulator of the initiation of X-chromosome inactivation in female placental mammals (Augui et al., 2011; Rastan and Brown, 1990), harboring the noncoding RNA *Xist* locus and the regulatory elements necessary for its female-specific developmental control. *Xist* is repressed in mouse embryonic stem cells (mESCs) and becomes upregulated from one of the two X-chromosomes in females upon exit of the pluripotent state, leading to random X-inactivation. This upregulation depends on the *Xist cis*-regulatory landscape (Heard et al., 1999), the full extent of which is still undefined; however, it is partitioned in at least two TADs, with the *Xist* locus lying close to the boundary between them (Fig. 1A) (Nora et al., 2012). The TAD in which the *Xist* promoter is included (referred to here as the Xist-TAD) contains some *Xist*-positive regulators (Augui et al., 2007; Barakat et al., 2011, 2014; Furlan et al., 2018; Gontan et al., 2012; Jonkers et al., 2009; Tian et al., 2010), whereas the adjacent TAD (referred to here as the Tsix-TAD) contains the promoter of *Tsix*, the antisense transcription unit to *Xist* that blocks its upregulation (Lee and Lu, 1999; Luikenhuis et al., 2001; Stavropoulos et al., 2001), as well as other elements that act as a *cis*-repressor of *Xist* [such as *Linx* and *Xite* (*Rr18*); see below].

To investigate how the topological organization of the Tsix-TAD impacts the regulation of genes both therein and in the neighboring Xist-TAD, we generated a mutant allele in mESCs and in mice with an inversion of almost the entire Tsix-TAD (245 kb out of 300 kb). We found that rewiring the Tsix-TAD structural landscape led to the formation of new chromatin contacts within the TAD, generally following the folding principles determined by the orientations of CTCF motifs. These topological alterations were accompanied by changes in gene expression of two out of seven genes within the TAD in differentiating mESCs. Interestingly, we found that the expression of *Xist* in the neighboring TAD was ectopically upregulated, suggesting that inter-TAD communication was affected.

## RESULTS

### Generating a genomic inversion encompassing the Tsix-TAD (245 kb-INV)

The Tsix-TAD harbors three hotspots of physical contacts (Fig. 1A), involving three different loci: (1) the *Xite* element, a proximal enhancer of *Tsix* (Ogawa and Lee, 2003), also involved in the position and insulation of the boundary between the Tsix- and Xist-TADs (van Bemmel et al., 2019); (2) the noncoding *Linx* locus, which harbors two *cis*-regulatory elements involved in controlling *Xist* expression (Galupa et al., 2020); and (3) *Chic1*, previously implicated in the maintenance of the organization of the Tsix-TAD (Giorgetti et al., 2014). Each of these loci harbors a set of CTCF sites involved in mediating the observed physical contacts and, within each locus, most CTCF motifs present the same orientation (Fig. 1A). Sites within *Linx* are ‘convergently oriented’ toward those within *Chic1* or *Xite*, the preferred orientation to form chromatin loops (Rao et al., 2014; Tang et al., 2015). Contacts between *Chic1* and *Xite* (the CTCF motifs of which occur in a ‘tandem’) are also observed (Fig. 1A). The contacts between these three loci might occur in pairwise fashion and/or simultaneously; physical modeling suggests that all conformations are possible (Giorgetti et al., 2014) and deletions of the CTCF-binding sites in either *Xite* (van Bemmel et al., 2019) or *Linx* (Galupa et al., 2020) show that contacts between the two remaining loci still occur.

We investigated whether this complex topological organization might be crucial for correct communication between the surrounding *cis*-regulatory elements (such as those within *Xite* and *Linx*) and their targets, therefore ensuring appropriate gene expression of *Tsix* and *Xist* and correct patterns of X-inactivation. Using a CRISPR/Cas9 editing approach in male mESCs, which carry a single X chromosome, we targeted a ∼245 kb region encompassing all loci within the Tsix-TAD, including the CTCF clusters within *Linx* and *Chic1*, but excluding *Xite* and *Tsix* (Fig. 1B). We decided not to include *Xite* in the inversion because: (1) *Xite* is already known to influence *Xist* expression (via *Tsix*); and (2) if *Xite* was inverted along with the rest of the TAD, the relative CTCF orientations between *Xite*, *Linx* and *Chic1* would not have changed. The targeted region does not involve either of the two boundaries of the TAD. We successfully generated two clones harboring an inversion allele (245 kb-INV) (Fig. 1C). This genomic inversion swaps the orientations of all CTCF motifs therein relative to those outside of the inverted region, in particular for *Linx* and *Chic1* (Fig. 1B) and, therefore, is expected to lead to the formation of new contacts within the TAD.

### 245 kb-INV leads to rearrangement of contacts within the TAD and increased insulation with neighboring TAD

**Fig. 2.**
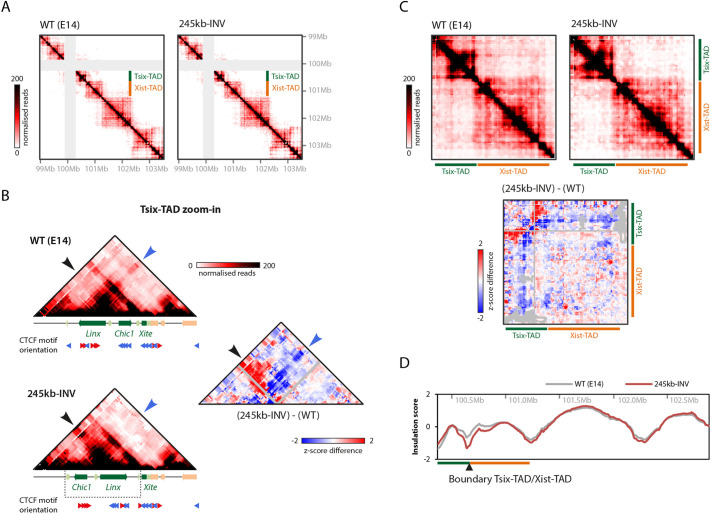
**Rearrangement of contacts within the TAD and increased insulation with neighboring TAD following 245** **kb intra-TAD inversion.** (A) 5C profiles of wild-type (WT; two replicates pooled) and 245 kb-INV mutant (two clones pooled) mESCs. The mutant map is corrected for inversion and gray pixels represent filtered contacts (see Materials and Methods). (B) Detailed view of the Tsix-TAD. The small red and blue arrowheads indicate the orientation of the CTCF motif (orientated left and right, respectively). The large black and large blue arrowheads indicate specific contacts that are gained or lost in the mutant, respectively (more details in the text). Gray pixels represent filtered contacts. (C) View of the Tsix- and Xist-TADs, and differential map representing the subtraction of *z*-scores calculated for WT and 245kb INV mutant maps separately. Gray pixels represent filtered contacts. (D) Insulation scores across the *Xic* TADs and downstream TADs based on 5C profiles for WT and 245 kb-INV mutant mESCs. The ‘troughs’ represent TAD boundaries.

To assess the topological organization of the 245 kb-INV allele, we performed carbon-copy chromosome conformation capture (5C) on the *Xic* (Dostie et al., 2006; Nora et al., 2012) for mutant and control mESCs (Fig. 2A). 5C analysis revealed that three hotspots of contacts can still be observed in the Tsix-TAD on the 245 kb-INV allele (Fig. 2B; please note that the 5C map is shown after ‘correction’ of the new genomic sequences in the inverted allele). These involve the same three loci as in control cells: in its new position, the *Chic1* CTCF cluster is able to establish contacts with *Linx* and with *Xite* (Fig. 2B); *Linx* and *Xite*, with CTCF sites in ‘tandem’ orientation in the 245 kb-INV allele, also interact (as *Chic1* and *Xite* do in control cells) (Fig. 2B). Therefore, inverting the *Linx* and *Chic1* CTCF clusters simultaneously appears to lead to new but similar hotspots of physical contacts within the Tsix-TAD compared with control. This might have been expected given that the overall distribution and orientation of CTCF sites within the TAD remain similar between the wild-type and the inverted alleles (Fig. 1B). In other words, the *Chic1* CTCF cluster on the inverted allele occupies an equivalent position to *Linx* on the wild-type allele, and vice versa. Therefore, the 245 kb inversion can lead to the formation of similar loops within the Tsix-TAD compared with the wild type, although involving different *cis*-regulatory elements.

Nevertheless, we also noticed some significant differences in the topology of the ‘inverted’ Tsix-TAD. Increased contacts were observed upstream of the inverted region, corresponding to contacts stemming from the *Linx* CTCF cluster in its new position (Fig. 2B, bottom, black arrow, red region in the differential map; this region shows no particular chromatin signatures, such as CTCF-binding or active chromatin marks). This suggests a different ‘strength’ for the *Linx* and *Chic1* CTCF clusters: in the inverted allele, the *Linx* CTCF cluster strongly interacts with regions upstream of *Chic1* (Fig. 2B, bottom, black arrow), whereas, in the wild-type configuration, the *Chic1* CTCF cluster does not form such strong contacts with regions upstream of *Linx* (Fig. 2B, top, black arrow). Conversely, we also observed a strong localized reduction in contacts (Fig. 2B, differential map, blue arrow) associated with the switch in positions between *Linx* and *Chic1*: the *Linx* CTCF cluster at its original position was able to form long-range contacts beyond *Chic1* and *Xite*, namely with elements within the Xist-TAD (Fig. 2B, top, blue arrow). These contacts were lost (or strongly reduced) in the 245 kb-INV cells (Fig. 2B, bottom and differential map, blue arrows), indicating that the *Chic1* CTCF cluster did not establish long-range contacts with the Xist-TAD when placed in the *Linx* CTCF cluster position. In fact, this loss of contacts across the boundary extended along the whole Xist-TAD (Fig. 2C). Again, this suggests a stronger potential for the *Linx* CTCF cluster to form contacts compared with the *Chic1* CTCF cluster.

We also evaluated the extent to which these topological changes had an impact on the overall insulation of the TADs. Insulation score analysis (see Materials and Methods) revealed a clear gain of insulation across the boundary between the Tsix-TAD and the Xist-TAD (Fig. 2D; lower insulation scores are reflective of increased insulation). The loss of *Linx*-mediated contacts across the boundary probably accounts, at least partially, for this increased insulation between the TADs. In summary, the 245 kb inversion repositions CTCF clusters within the Tsix-TAD, leading to reconfiguration of specific intra- and inter-TAD contacts accompanied by stronger insulation with the neighboring Xist-TAD.

### 245 kb-INV leads to gene expression changes within the Tsix-TAD and across the boundary

**Fig. 3.**
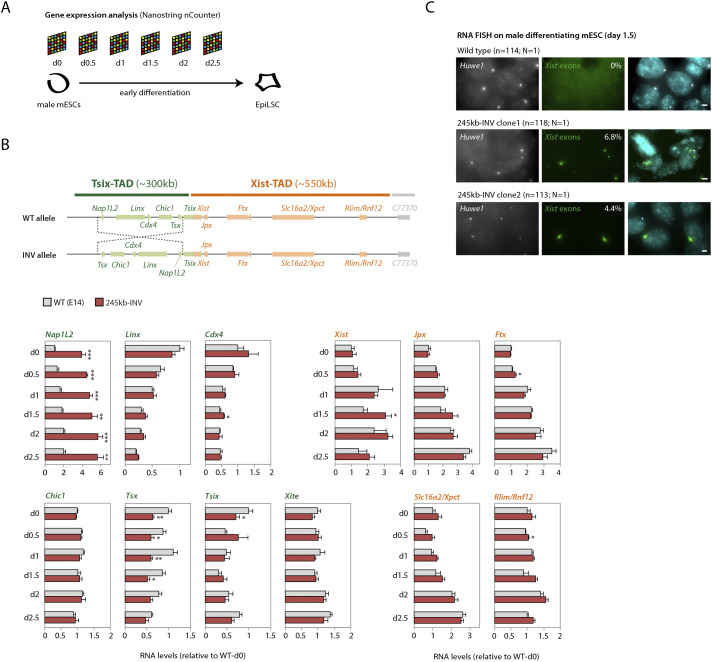
**Inversion leads to transcriptional changes of specific genes within the TAD and of *Xist* across the TAD boundary.** (A) Schematic of the mESC-to-epiblast-like stem cell (EpiLSC) differentiation and time points analyzed by Nanostring nCounter (see Materials and Methods). (B) Gene expression analysis during differentiation (d0-d2.5). Data are normalized to wt-d0 for each gene, and represent the mean±s.d. of two biological replicates (wild type) or of two independent clones (mutant). Data were analyzed with a two-tailed paired Student's *t*-test (*P<0.05; **P<0.01; ***P<0.001). (C) RNA FISH for *Huwe1* (X-linked gene outside of the *Xic*) and *Xist* (exonic probe) on mESCs differentiated to d1.5. The percentage of cells with *Xist* RNA accumulation is indicated and represents the mean from two independent experiments, where *N* indicates the number of experiments and *n* indicates the number of nuclei counted. Scale bars: 2 µm.

We next set out to determine whether similar interaction patterns, but different wiring of sequences within the Tsix-TAD, led to any transcriptional changes. To this end, we profiled transcript levels across the *Xic* using digital gene expression analysis (NanoString) (Geiss et al., 2008) in control and mutant cells in the pluripotent state (d0) and during early differentiation (d0.5-d2.5) (Fig. 3A). Expression of most genes within the Tsix-TAD and the Xist-TAD was unaffected in 245 kb-INV cells (Fig. 3B), including that of the three loci involved in the topological alterations: *Linx*, *Chic1* and *Xite*. This suggests no or limited effect of the structural alterations on the transcriptional regulation of these loci. Expression of *Tsix* was significantly reduced in mutant cells in the pluripotent state (d0) (Fig. 3B), but this effect did not persist consistently during differentiation. The deletion of the same region that we inverted here also led to downregulation of *Tsix* in mESCs (Galupa et al., 2020); together with the current data, this suggests that the region contains important sequences for *Tsix* regulation, that this regulation depends on the orientation of the region as a whole and might also depend on the orientation of individual regulatory sequences.

However, we did notice consistent changes during differentiation in mutant cells for two genes within the Tsix-TAD: *Nap1l2*, which was significantly upregulated at all time points (Fig. 3B) and *Tsx*, which was significantly downregulated (Fig. 3B). Interestingly, both genes lie at the extremities of the inverted region, and switch their relative positions in the TAD between the wild-type and mutant configurations. It is likely that changes in their gene expression are associated with altered proximity to the *Xite* enhancer element. Given that deletion of *Xite* leads to downregulation of *Tsx* (van Bemmel et al., 2019), moving *Tsx* away from *Xite* on the 245 kb-INV allele could lead to its observed downregulation. Conversely, the increased linear proximity of *Nap1l2* to *Xite* could underlie *Nap1l2* upregulation. Changes in interaction frequencies between *Xite* and these two elements in the 245 kb-INV allele support this hypothesis, because they reflect the changes in their genomic distances (increased for *Xite-Nap1l2* and decreased for *Xite-Tsx*, compared with control; Fig. S1).

We also observed changes in expression of *Xist*, the long noncoding RNA locus that is regulated by the *Xic* to trigger the initiation of X-chromosome inactivation. Normally very low in male cells, *Xist* expression was slightly upregulated in the mutants at later differentiation time points (∼twofold at d1.5; Fig. 3B). In female cells undergoing X-inactivation, upregulation of *Xist* is accompanied by local accumulation of its RNA in *cis*, forming a so-called ‘Xist cloud’, which can readily be detected by RNA fluorescence *in situ* hybridization (FISH) (Augui et al., 2011). RNA FISH revealed the formation of *Xist* clouds in ∼4-7% of mutant male cells upon differentiation, which was never observed in wild-type male cells (Fig. 3C). Thus, the inversion of 245 kb within the Tsix-TAD leads to ectopic expression of *Xist*, the promoter of which is located in the neighboring TAD.

### Female embryos with a 245 kb-INV allele show higher *Xist* allelic imbalance

**Fig. 4.**
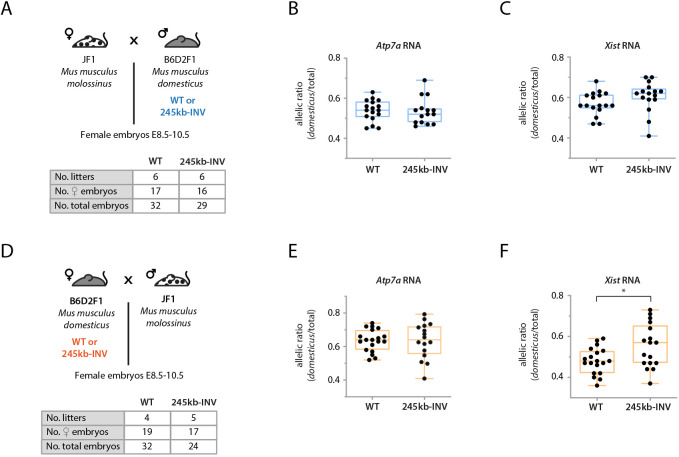
**Female embryos with the 245** **kb-INV allele show a bias in *Xist* expression.** (A,D) Crosses used for the analysis of RNA allelic ratios in female hybrid embryos inheriting the *Mus musculus domesticus* allele either paternally (A; blue) or maternally (D; red). Tables summarize the number of embryos collected. (B,C,E,F) RNA allelic ratios for the X-linked gene *Atp7a* (B,E) and *Xist* (C,F). Each black dot corresponds to a single female embryo. Box-and-whisker plots indicate median, interquartile range and min/max values, respectively, with blue and red plots indicating paternally or maternally inherited alleles, respectively. Data were analyzed using the Mann–Whitney U test (*P<0.05).

Given the impact of the 245 kb inversion on *Xist* expression in male cells, we investigated whether this was also the case in female embryos at post-implantation stages, when random X-inactivation is known to have already occurred (Rastan, 1982). To this end, we generated an equivalent 245 kb-INV allele in mice (see Materials and Methods) and collected post-implantation heterozygous embryos. These embryos were derived from crosses between polymorphic mouse strains (Fig. 4A,D), which allowed us to distinguish the allelic origin of transcripts. Analysis of RNA allelic ratios for *Atp7a*, an X-linked gene, revealed no preferential gene silencing for one or the other allele (Fig. 4B,E).

However, analysis of *Xist* RNA allelic ratios between mutant and control embryos showed slightly higher *Xist* allelic ratios in the mutant embryos, regardless of whether the mutant allele was inherited paternally (Fig. 4C) or maternally (Fig. 4F); this increase was statistically significant for maternal transmission (*P*<0.05). These results are consistent with the upregulation of *Xist* that we observed in mutant cells (Fig. 3B,C). Thus, the 245 kb inversion leads to higher *Xist* levels in *cis*, but this does not result in skewed patterns of X-inactivation. Of note, litter size appeared to be reduced upon maternal transmission of the 245 kb-INV allele, with a skewed sex ratio (71% females in 245 kb-INV versus 59% in control), suggesting that the inversion may have more phenotypic consequences.

### Mutating clusters of CTCF sites within *Linx* and *Chic1* lead to changes in *Xist* expression

**Fig. 5.**
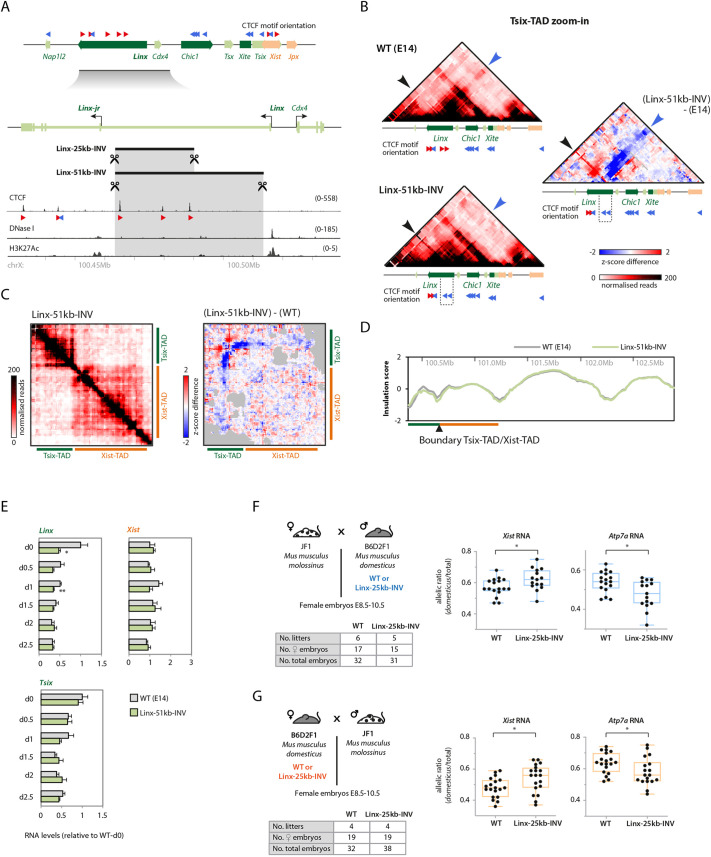
**Inversion of *Linx* cluster of CTCF sites leads to Xist upregulation in *cis*.** (A) The *Linx* locus, CTCF binding, and orientation of CTCF motifs associated with CTCF ChIP-seq peaks. The red and blue arrowheads indicate the orientation of the CTCF motif (orientated left or right, respectively). The targeted inversions *Linx*-25 kb-INV and *Linx*-51 kb-INV are indicated. (B) 5C profiles (Tsix-TAD zoom-in) of wild-type (WT; two replicates pooled) and *Linx*-51 kb-INV (two clones pooled) mESCs, and 5C differential map, representing the subtraction of *z*-scores calculated for WT and Linx-51 kb-INV maps. The large black and large blue arrowheads indicate specific contacts that are gained or lost in the mutant, respectively (more details in the text). (C) (Left) 5C profile of Linx-51 kb-INV mESCs (two clones pooled); the map is corrected for inversion and gray pixels represent filtered contacts (see Materials and Methods); (Right) 5C differential map, representing the subtraction of *z*-scores calculated for WT and *Linx*-51 kb-INV maps separately. (D) Insulation scores across the *Xic* TADs and downstream TADs based on 5C profiles for WT and *Linx*-51 kb-INV mESCs. The ‘troughs’ represent TAD boundaries. (E) Gene expression analysis during differentiation (d0-d2.5). Data are normalized to wt-d0 for each gene, and represent the mean±s.d. of two biological replicates (WT) or of two independent clones (mutant). Data were analyzed with a two-tailed paired Student's *t*-test. (F,G) (Left) Crosses used for analysis of RNA allelic ratios in female hybrid embryos inheriting the *Mus musculus domesticus* allele either paternally (F; blue) or maternally (G; red). Tables summarize the number of embryos collected. (Right) RNA allelic ratios for *Xist* and the X-linked gene *Atp7a*. Each black dot corresponds to a single female embryo. Box-and-whisker plots indicate median, interquartile range and min/max values, with blue and red plots indicating paternally or maternally inherited alleles, respectively. Data were analyzed using the Mann–Whitney U test (*P<0.05; **P<0.01).

To further explore the link between the topological organization of the Tsix-TAD and *Xist* regulation, we generated alleles with deletions and/or inversions of the clusters of CTCF sites within *Linx* and within *Chic1*. We previously deleted a large intronic interval containing three *Linx* CTCF sites from male ESCs (∼51 kb) and from mice (∼25 kb) (Galupa et al., 2020), which led to some alterations in the topological organization of the two *Xic* TADs but no changes in *Xist* expression in female embryos. Thus, we tested the impact of inversions of exactly the same regions in male mESCs (Linx-51 kb-INV) and in mice (Linx-25 kb-INV) (Fig. 5A). 5C analysis of the Linx-51 kb-INV allele revealed higher frequency of contacts between the now-inverted *Linx* locus and regions immediately upstream (Fig. 5B, black arrowhead), and lower frequency of contacts between (inverted) *Linx* and *Chic1* and between *Xite* and elements within the Xist-TAD (Fig. 5B,C, blue arrowhead), in agreement with the change in orientation of the three *Linx* CTCF sites. These results are reminiscent of what we observed for the 245 kb-INV allele (Fig. 2B,C), and support the hypothesis that loss of contacts with the Xist-TAD in the 245 kb-INV allele is associated with inversion of the CTCF sites within *Linx*. Consistently, analysis of insulation scores across the TADs revealed a gain of insulation across the boundary between the Tsix-TAD and the Xist-TAD (Fig. 5D), although less pronounced than that observed for the 245 kb-INV allele (Fig. 2D). We next analyzed gene expression across the *Xic* for the Linx-51 kb-INV mESCs in the pluripotent state (d0) and during early differentiation (d0.5-d2.5); expression of *Linx* was significantly downregulated at some time points (Fig. 5E) but no changes were observed for *Xist* or *Tsix* (Fig. 5E) or for any other locus across the *Xic* (Fig. S2). However, when we analyzed *Xist* expression in female embryos carrying an heterozygous *Linx*-25 kb-INV allele, we observed significantly higher expression of *Xist* for the inverted allele, for both paternal and maternal transmission (Fig. 5F,G), and also a corresponding decrease in expression of the X-linked gene *Atp7a* (Fig. 5F,G), suggestive of skewed X chromosome inactivation compared with control. Overall, the inversion of the *Linx* CTCF cluster led to similar phenotypes compared with the large 245 kb inversion, namely a decrease in contact frequency between *Linx* and the Xist-TADs, a concomitant gain of insulation between them, and increased *Xist* expression in *cis* in female embryos.

**Fig. 6.**
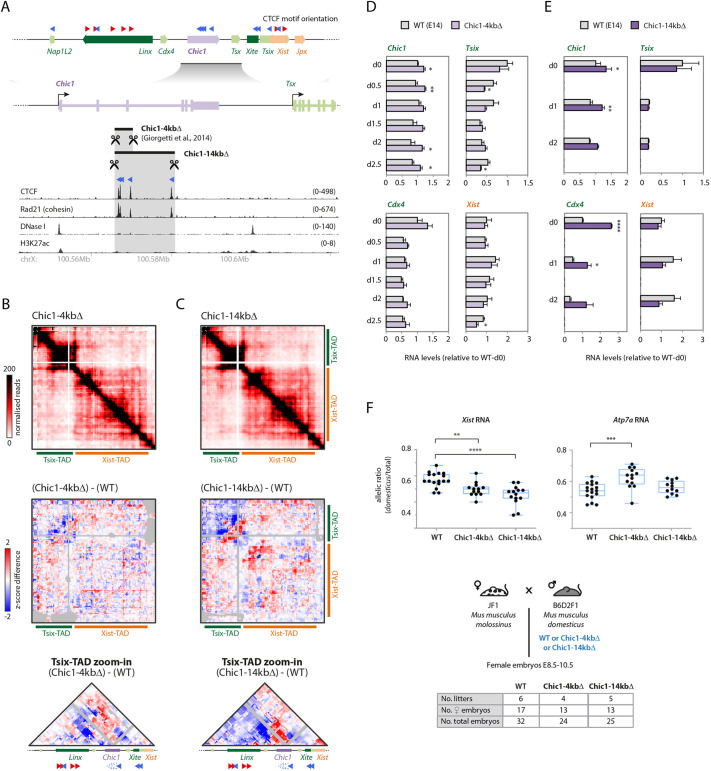
**Deletion of *Chic1* cluster of CTCF sites leads to *Xist* downregulation in *cis*.** (A) The *Chic1* locus, CTCF binding and orientation of CTCF motifs associated with CTCF ChIP-seq peaks. The targeted deletions *Chic1*-4 kbΔ and *Chic1*-14 kbΔ are indicated. The red and blue arrowheads indicate the orientation of the CTCF motif (orientated left or right, respectively). (B,C) (Top) 5C profiles of *Chic1*-4 kbΔ (B, two clones pooled) and *Chic1*-14 kbΔ (C, one clone, two replicates pooled). (Middle) 5C differential maps, representing the subtraction of *z*-scores calculated for wild-type (WT) and deletion maps. Gray pixels represent filtered contacts (see Materials and Methods). (Bottom) Differential maps for the Tsix-TAD. (D,E) Gene expression analysis during differentiation (d0-d2.5). Data are normalized to wt-d0 for each gene, and represent the mean±s.d. of two biological replicates (WT and *Chic1*-14 kbΔ) or of two independent clones (*Chic1*-4 kbΔ). Data were analyzed with a two-tailed paired Student's *t*-test (*P<0.05; **P<0.01; ****P<0.0001). (F) (Top) RNA allelic ratios for *Xist* and the X-linked gene *Atp7a*. Each black dot corresponds to a single female embryo. Box-and-whisker plots indicate median, interquartile range and min/max values. Data were analyzed with a Mann–Whitney U test (**P<0.01; ***P<0.001; ****P<0.0001). (Bottom) Crosses used for the analysis of RNA allelic ratios in female hybrid embryos inheriting the *Mus musculus domesticus* allele paternally. Tables summarize the number of embryos collected.

We previously generated in male mESCs a ∼4 kb deletion within *Chic1* (Giorgetti et al., 2014) that encompassed two of the three CTCF-binding sites present in the locus (Fig. 6A), but we did not study its impact on chromosome conformation or on *Xist* expression, which we set out to do here. Differential 5C analysis between this *Chic1*-4 kbΔ allele and wild type showed a reduction in contacts between *Chic1* and *Linx* and also between *Chic1* and *Xite* (Fig. 6B), consistent with loss of the *Chic1* CTCF sites. We also noted an apparent increase in contact frequency between *Linx* and *Xite* (Fig. 6B; Fig. S1), which would be consistent with a model of competition between *Chic1* and *Xite* CTCF sites to form loops with the CTCF sites within *Linx*. However, these differences in contact frequencies overall remained rather close to the ‘noise’ levels of the 5C map. We wondered whether these effects would be more pronounced if the remaining CTCF-binding site was also removed; thus, we generated, in male mESCs, a larger deletion (*Chic1*-14 kbΔ) encompassing all three CTCF sites within *Chic1* (Fig. 6A). We observed more-pronounced contact rearrangements within the Tsix-TAD as for *Chic1*-4 kbΔ (Fig. 6C), suggesting that it is the loss of the CTCF sites that underlies the observed topological differences. To study the impact of these deletions on gene expression across the *Xic*, we profiled transcript levels, as performed previously, in the pluripotent state (d0) and during early differentiation. Expression of *Chic1* itself was consistently upregulated in both *Chic1*-4 kbΔ and *Chic1*-14 kbΔ (Fig. 6D,E); it is intriguing to think that this could be linked to its now shorter length, because shorter genes have been associated with higher levels of expression (Castillo-Davis et al., 2002; Chiaromonte et al., 2003). We also observed higher expression of *Cdx4*, the gene upstream of *Chic1*: interestingly, the effects appeared to scale up with the larger deletion: in *Chic1*-4 kbΔ mESCs, there was a slight increase in *Cdx4* levels across time points but this was not statistically significant, whereas, in *Chic1*-14 kbΔ, the increase was more pronounced and statistically significant for some time points. This effect could be connected to the removal of all CTCF sites from within the *Chic1* locus, which could ‘shield’, or insulate, *Cdx4* from activating influences downstream of the CTCF sites. *Xist* expression was also more affected in mESCs containing the larger deletion: we observed a mostly consistent downregulation across all time points, but this effect was not statistically significant in this context. However, in female embryos, we did observe a statistically significant decrease in *Xist* expression from the deletion alleles (Fig. 6D,E), which was more pronounced for the *Chic1*-14 kbΔ allele. This suggests that the *Chic1* CTCF cluster might operate to favor *Xist* expression in *cis*. These results potentially also illustrate how loss of one additional CTCF-binding site might be enough to cause stronger changes in chromosome conformation and gene expression.

Together, our results on inverting or deleting *Linx* and *Chic1* CTCF clusters highlight the rather complex regulatory landscape within the *Xic*. Similar to the 245 kb inversion, these mutant alleles reveal how *Xist* is sensitive to changes involving CTCF-binding sites within the neighboring Tsix-TAD. These results also suggest that the phenotypes observed in the 245 kb-INV mESCs are likely a combination of effects from changing different elements within the Tsix-TAD.

## DISCUSSION

Here, we explored the structural and transcriptional consequences of inverting a large genomic region encompassing almost an entire TAD (80%; 245 kb out of 300 kb). We found that this inversion led to rearrangement of contacts and to changes in expression of some genes within the TAD. We also observed increased contact insulation with the neighboring TAD and ectopic upregulation of a gene in that TAD, the noncoding RNA *Xist* locus.

The rearrangement of contacts within the Tsix-TAD upon inverting a 245 kb region occurred largely as expected based on the ‘rules’ associated with the orientation of CTCF motifs within the TAD (de Wit et al., 2015; Guo et al., 2015; Rao et al., 2014; Sanborn et al., 2015; Tang et al., 2015). We found that the three loci involved in the strong contacts observed in the wild-type Tsix-TAD were still able to form strong contacts with each other in the ‘inverted’ Tsix-TAD (Fig. 2). Yet, these elements could not fully replace each other in their new positions, despite similar composition in terms of number of CTCF sites and levels of CTCF binding based on published chromatin immunoprecipitation-sequencing (ChIP-seq) data (Fig. 1). In particular, the *Linx* CTCF cluster appears to have a stronger potential to form contacts compared with the *Chic1* CTCF cluster. At the same relative position within the TAD, and with the same CTCF motif orientation, these CTCF clusters show a different range of interactions, as described above (Fig. 2). These differences suggest that not all CTCF-bound sites are equally capable of mediating the same type of interactions. Little is known about what determines which CTCF sites contact with each other, and whether there could be specific affinities between sites depending, for instance, on which other protein complexes are bound at each site or nearby. The ChIP-seq signal for CTCF is comparable between CTCF sites within *Linx* and *Chic1*, and there are the same number of CTCF sites within each locus. One difference in the organization of these sites is the spacing between them: CTCF sites within *Chic1* are more clustered than are those within *Linx*; this could play a role in orchestrating which contacts are formed, and how. ‘Loop extrusion’ has been proposed as a mechanism to form TADs and chromatin contacts (Fudenberg et al., 2016; Goloborodko et al., 2016; Sanborn et al., 2015), by which an ‘extruding factor’ (such as cohesin) engulfs two DNA chains and moves along them, extruding DNA until it reaches ‘stalling factors’ (such as CTCF), which block its progression; thus, a chromatin loop would be formed and stabilized. Could the length of the intervals between CTCF sites influence the likely point at which the cohesin complex (or the extruding factor) gets stalled? Perhaps more-distributed sites (as at the *Linx* locus) rather than more-clustered sites (as at the *Chic1* locus) provide more opportunities for stalling cohesin, given the very fast rate at which CTCF binds and unbinds chromatin (Hansen et al., 2017) and the rate of extrusion by cohesin (Davidson et al., 2019; Kim et al., 2019). Another potential explanation (not mutually exclusive) is that the differences in ‘contact potential’ depend on the different sequences flanking the consensus CTCF motifs within *Linx* and *Chic1*, as suggested by a recent study of CTCF sites as transcriptional insulators (Huang et al., 2021).

Transcriptional changes in mutant 245 kb-INV mESCs and during differentiation were observed for two genes (*Nap1l2* and *Tsx*) within the Tsix-TAD when compared with controls (Fig. 3). As discussed previously, we suggest that these changes are associated with (genomic) proximity to the enhancer element *Xite* and not necessarily with the new topological structure of the inverted Tsix-TAD. Perhaps more interesting is the fact that most other genes within the Tsix-TAD do not show changes in expression, especially the *Linx*, *Chic1* and *Xite* loci, which are involved in the topological changes observed for the 245 kb-INV allele. This could have a number of explanations: (i) the expression of these genes might not be particularly reliant on *cis*-regulation and, therefore, might be impervious to topological changes; (ii) interactions between these genes and their *cis*-regulatory elements might not depend on topological organization and, therefore, still occur regardless of the topological changes; or (iii) interactions between these genes and their *cis*-regulatory elements might depend on the topological organization; the new contacts allow these interactions to occur as efficiently as in wild type and, therefore, no changes in expression are observed. Further genetic exploration of these loci will be crucial to exclude hypotheses.

Surprisingly, expression of *Xist*, which lies outside of the Tsix-TAD, in the neighboring TAD, was mildly upregulated, to an extent that we could detect accumulation of Xist RNA in ‘clouds’ in mutant male cells, which we never observe(d) in wild-type male cells (Fig. 3). This upregulation could be associated with one or more of the other alterations observed on the 245 kb-INV allele, either structural, or transcriptional, or both. For instance, we observed reduced expression of *Tsix*, the antisense *cis*-repressor of *Xist*, in the pluripotent state, which could impact *Xist* regulation; during differentiation, however, when *Xist* is upregulated, we did not detect differences in *Tsix* expression. Further research will be needed to clarify the involvement of *Tsix* in the *Xist* phenotype observed here.

Could *Xist* upregulation be a consequence of *Nap1l2* upregulation or *Tsx* downregulation? Genetic studies with *Nap1l2* (Attia et al., 2007; Rogner et al., 2000) did not report any effects on *Xist* expression or sex-specific phenotypes; thus, upregulation of *Nap1l2* is unlikely to cause *Xist* upregulation, although this cannot be formally excluded. By contrast, knockout studies of *Tsx* (Anguera et al., 2011) reported *Xist* RNA clouds in a small percentage of differentiating male mutant mESCs; the authors proposed that *Xist* expression was upregulated because of its negative *cis*-regulators *Tsix* and *Xite* being downregulated during differentiation. This *Xist* phenotype is identical to the one we observed (Fig. 3), although, in 245 kb-INV mutant cells, there is still some *Tsx* expression (contrary to the *Tsx* knockout) and we did not observe changes in *Tsix* or *Xite* expression during differentiation. Thus, downregulation of *Tsx* in 245 kb-INV mutant cells might account, partially or perhaps even completely, for ectopic *Xist* upregulation. This raises interesting questions of how such inter-TAD regulation/communication between *Tsx* and *Xist* could occur. Similarly, we recently reported that another locus within the Tsix-TAD, *Linx*, contains sequences that affect expression of *Xist* in the neighboring TAD in a *Tsix*-independent manner (Galupa et al., 2020). A slight increase in *Xist* expression in *cis* was also observed in 245 kb-INV heterozygous embryos, but it was not statistically significant and did not result in skewed patterns of X-inactivation (Fig. 4). These results underlie the importance of verifying whether changes in gene expression result in differences in the phenotypes they mediate: in many studies, it often remains an open question whether the changes observed in gene expression, especially when modest, do matter for the processes in which those genes are involved.

In agreement with previous studies, our study illustrates that the relationship between chromosome structure and gene expression is rather complex. The almost ‘dogmatic’ view that TADs restrict gene *cis*-regulation (Finn and Misteli, 2019; Koch, 2019) is at odds with a growing amount of evidence that mechanisms of inter-TAD communication exist, albeit potentially subject to modulation by TADs and their boundaries. Here, we showed that, on the one hand, expression of genes within a TAD can be tolerant to changes in contacts within that TAD, whereas, on the other hand, inversion of a large region within a TAD affected the expression of a gene in the neighboring TAD, potentially because of accompanying changes in topological organization and topological insulation. Further investigations are warranted for a more-complete understanding of the relationship between the topological organization of the genome and the transcriptional regulation of its genes.

## MATERIALS AND METHODS

All the materials and methods described below have also been published previously (Galupa et al., 2020).

### Tissue culture conditions

The E14 mESC line and clones derived from it were grown in flasks or on dishes coated with 0.1% (wt/vol) gelatin. Culture media for mESCs comprised Glasgow medium (Gibco) supplemented with 2 mM L-glutamine, 0.1 mM nonessential amino acids, 1 mM sodium pyruvate, 15% fetal bovine serum (FBS) (Gibco), 0.1 mM b-mercaptoethanol (Sigma) and 1000 U/ml leukemia inhibitory factor (LIF) (Chemicon). All lines were cultivated at 37°C under 8% CO_2_ and passaged according to their confluency, generally every other day. Medium was refreshed daily. For early EpiLSC differentiation assays, mESCs were washed with 1× PBS, incubated with trypsin at 37°C for 20 min and resuspended in ES medium without LIF. After cell counting, the desired number of cells was resuspended in differentiation medium and 8×10^5^ cells per well were seeded on a fibronectin-coated (10 µg/ml, Millipore) six-well plate in differentiation medium. Differentiation medium comprised N2B27 medium, 20 ng/ml activin A (R&D Systems) and 12 ng/ml FGF-basic (R&D Systems). Differentiation medium was changed daily and cells were washed in PBS before collection to remove dead cells. Cells were routinely checked for mycoplasma contamination.

### Mouse experimentation

Animal care and use for this study were performed in accordance with the recommendations of the European Community (2010/63/UE) for the care and use of laboratory animals. Experimental procedures, including genomic engineering (see below), were in compliance with international guidelines and were specifically approved by the ethics committee of the Institut Curie CEEA-IC #118 and given authorization by the French national authorities (references: APAFIS##13962-2018030717538778-v2 and APAFIS#8812-2017020611033784-v2).

Postimplantation embryos were collected at the embryonic day (E) 8.5-10.5 stage, assuming plugging at midnight. Females with a vaginal plug were weighed every other day and only taken for dissection if a significant increase in weight was observed (∼2 g for B6D2F1 mice, ∼1 g for JF1 mice) at the expected point of E8.5-E10.5 development. Extra-embryonic tissues were taken for sexing the embryos. Whole embryos were washed three times in 1× PBS before being frozen for allelic expression analysis.

### Genomic engineering of mice and mESCs

Inversion 245 kb-INV and deletion *Chic1*-14 kbΔ were generated using CRISPR-Cas9 (mESCs and mice) technologies, using the process described below. Inversions within the *Linx* locus (*Linx*-25 kb-INV and *Linx*-51 kb-INV) were generated using the same constructs and primers as the equivalent deletions, described in Galupa et al. (2020). The *Chic1*-4 kbΔ deletion had been generated previously (Giorgetti et al., 2014).

We designed short guide (sg) RNAs to flank the region of interest:

- For 245 kb-INV: CR30 (ACTGGTTCAGCCACTCACCG) and CR32 (CTGAGCTGGTTCATACAGGT).
- For *Chic1*-14 kbΔ: CR21 (AAAGATCGTTTCTATCTAGC) and CR16R (CGCCAAACTTCCAAAATGGC).

For cloning sgRNAs, we used pX459-v2 (Addgene 62988) and a protocol from the Zhang lab (https://media.addgene.org/cms/filer_public/e6/5a/e65a9ef8-c8ac-4f88-98da-3b7d7960394c/zhang-lab-general-cloning-protocol.pdf). sgRNA constructs were amplified upon transformation of DH5α competent cells (Takara) grown at 37°C, and then sequenced to verify that the cloning was correct. Midipreps for all constructs were prepared at a final concentration >1 mg/ml using the NucleoBond Xtra Midi Plus kit (Macherey-Nagel).

mESCs were transfected with sgRNA constructs using the P3 Primary Cell 4D-Nucleofector X Kit (Lonza) and the Amaxa 4D Nucleofector™ system (Lonza), with the transfection program CG-104. Each transfection included 5 million cells resuspended in the nucleofection mix (prepared according to the manufacturer's instructions) containing 5 µg of each sgRNA (two constructs). As a transfection control, 10 µg of pmaxGFP (Lonza) was used, for which the nucleofection efficiency was around 90%. Cells were immediately resuspended in pre-warmed culture medium after nucleofection and seeded at three serial 10× dilutions in 10-cm dishes to ensure optimal density for colony picking. Transfected cells were selected with puromycin for 48 h, and grown for 8-10 days. Single colonies were picked into 96-well plates. Genomic DNA was isolated in 96-well plates for PCR-based screening of inversions. The genotyping primers used were as follows:

- For 245 kb-INV: RG82 (CAATCACTCTTGCCTTACCAATT), RG83 (CCCAAACCAACCCTTGACTG), RG84 (GTTGGGACCTAAACTCTAGTACA), RG85 (AGTGGACTAGCTTTGCCTCA).
- For *Chic1*-14 kbΔ: EN118 (GCCTGCAGTCTTACCAGGAG), EN119 (TAATCTGCAGCGTGTTGAGG), RG123 (TCCTCCCTTACCAGTCTCCT), RG124 (CAGAATCCCGGATGTGAGGA).

We sequenced the PCR products from the inversion alleles to determine the breakpoints:

- For 245 kb-INV: clone1, chrX-100377328 and chrX-100622017; clone2, chrX-100377337 and chrX-100622025 (coordinates in mm9).
- For *Chic1*-14 kbΔ: clone1, chrX-103370850 and chrX-103384956 (coordinates mm10).

The mouse mutant lines were generated following the strategy described by Wang et al. (2013) with minor modifications. Cas9 mRNA was transcribed *in vitro* from a T7-Cas9 pCR2.1-XL plasmid (Greenberg et al., 2017) using the mMESSAGE mMACHINE T7 ULTRA kit (Life Technologies) and purified with the RNeasy Mini kit (Qiagen), or bought from Tebu-bio (L-7206). The sgRNAs were amplified by PCR with primers containing a 5′ T7 promoter sequence from the plasmids used for mESC transfection. After gel purification, the T7-sgRNA PCR products were used as the template for *in vitro* transcription with the MEGAshortscript T7 kit (Life Technologies) and the products were purified using the MEGAclear kit (Life Technologies). Cas9 mRNA and the sgRNAs were eluted in DEPC-treated RNase-free water, and their quality was assessed by electrophoresis on an agarose gel after incubation at 95°C for 3 min with the denaturing agent provided with the *in vitro* transcription kits. Cas9 mRNA and sgRNAs (at 100 ng/μl and 50 ng/μl, respectively) were injected into the cytoplasm of mouse B6D2F1 zygotes from 8-week-old superovulated B6D2F1 (C57BL/6J×DBA2) females mated to stud males of the same background. Zygotes with well-recognized pronuclei were collected in M2 medium (Sigma) at E0.5. Injected embryos were cultured in M16 medium (Sigma) at 37°C under 5% CO_2_, until transfer at the one-cell stage the same day or at the two-cell stage the following day to the infundibulum of the oviduct of a pseudogestant CD1 female at E0.5 (25-30 embryos were transferred per female). All weaned mice (N0) were genotyped for the presence of inversion alleles using the same genotyping primers as for mESC mutant lines. Mice carrying inversion alleles were crossed to B6D2F1 mice and their progeny screened again for the presence of the inversion allele. The F1 mice were considered the ‘founders’ and bred to B6D2F1 mice; their progeny was then intercrossed to generate homozygous mice and lines were kept in homozygosity.

### RNA fluorescence *in situ* hybridization

RNA FISH was performed as described previously with minor modifications (Chaumeil et al., 2008). Briefly, differentiating mESCs were dissociated using accutase (Invitrogen) and adsorbed onto poly-L-lysine (Sigma)-coated coverslips #1.5 (1 mm) for 5 min. Cells were fixed with 3% paraformaldehyde in PBS for 10 min at room temperature and permeabilized for 5 min on ice in PBS containing 0.5% Triton X-100 and 2 mM ribonucleoside-vanadyl complex (New England Biolabs). Coverslips were preserved in 70% ethanol (EtOH) at −20°C. To begin the FISH experiments, coverslips were dehydrated through an EtOH series (80%, 95% and twice at 100%) and air dried quickly, then lowered onto a drop of the probe/hybridization buffer mix [50% formamide, 20% dextran sulfate, 2× saline-sodium citrate (SSC), 1 μg/μl BSA, 10 mM ribonucleoside-vanadyl complex] and incubated overnight at 37°C. The next day, coverslips were washed three times at 42°C in 50% formamide in 2× SSC (pH 7.2-7.4) and three times at 42°C in 2× SSC. Nuclei were counterstained with DAPI (0.2 mg/ml), coverslips were mounted [90% glycerol, 0.1× PBS, 0.1% p-phenylenediamine at pH 9], and cells were imaged using a wide-field DeltaVision Core microscope (Applied Precision).

The probes used were a *Huwe1* bacterial artificial chromosome (BAC; BACPAC Resources Center, RP24-157H12) and oligos (∼75 nucleotides long) covering all *Xist* exons (Roche, custom design). The BAC was labeled using the Nick Translation kit (Abbot) following the manufacturer's instructions. Oligos were end-labeled with an Alexa488 fluorophore (Abbot). Probes were either EtOH precipitated (BAC) or vacuum dried (oligos) and resuspended in formamide with shaking at 37°C. BAC was coprecipitated with mouse Cot-1 DNA (Invitrogen), and competition to block repetitive sequences was performed for at least 20 min at 37°C, and after denaturation (75°C, 10 min). Probes were then mixed with one volume of 2× hybridization buffer.

### Gene expression analysis (mESCs)

Cells were collected for gene expression analysis at 0 h, 12 h, 24 h, 36 h, 48 h and 60 h of EpiLSC differentiation. Cells were lysed with Trizol (Invitrogen), and RNA was isolated using the RNAeasy Mini kit (Qiagen), including DNase treatment. RNA samples were systematically run on an agarose gel to check their integrity. For reverse transcription, cDNA was synthesized from 0.5 μg of RNA using SuperScript™ III Reverse Transcriptase and random primers (both Invitrogen) according to the manufacturer's recommendations. Two independent reverse transcription experiments were carried out for each sample, pooled at the end and diluted 25-fold prior to qPCR or allelic expression analysis. No-reverse transcription controls were processed in parallel. The NanoString nCounter gene expression system (Geiss et al., 2008) was used to characterize transcriptional differences in wild-type and mutant mESCs systematically, before or during differentiation. We used 500 ng of total RNA from each sample for each nCounter hybridization round. We designed a customized probe codeset (van Bemmel et al., 2019) to identify nearly a hundred transcripts from *Xic* genes, other X-linked genes, pluripotency factors, differentiation markers, proliferation markers and normalization genes. Standard positive controls included in the kit were used for scaling the raw data. The genes *Actb*, *Rrm2* and *Sdha* were used for normalization. Differential expression was always calculated for samples run on the same nCounter hybridization.

### Allelic expression analysis (mouse embryos)

Embryos were lysed in RLT buffer (Qiagen) supplemented with 0.01% 2-mercaptoethanol. After two rounds of vortexing (15 s each), lysates were applied directly to a QIAshredder spin column (Qiagen) and centrifuged for 3 min at >15,000 ***g***. RNA was extracted using the RNAeasy Mini kit, including DNase treatment, and following the manufacturer's instructions. RNA samples were systematically run on an agarose gel to check their integrity. cDNA was prepared as described above for the gene expression analysis of mESCs, and was then PCR amplified with biotinylated primers and pyrosequenced for allele quantification on a Pyromark Q24 system (Qiagen). The same PCR approach was performed on no-reverse transcription control samples to confirm the absence of genomic DNA contamination. The primers used were designed with PyroMark Assay Design software and validated on XX polymorphic genomic DNA at a ratio of 50:50% (±4%). A list of primers and SNPs used for allele quantification can be found in Galupa et al. (2020).

### Chromosome conformation capture

3C libraries were prepared based on previous protocols (Nora et al., 2017; Rao et al., 2014), with some modifications. Crosslinked cells (in 2% formaldehyde; 10 million for each sample) were lysed in 10 mM Tris-HCl (pH 8), 10 mM NaCl, 0.2% NP-40, 1× complete protease inhibitor cocktail (Roche) for 15 min on ice. Nuclei were resuspended in 100 μl 0.5% SDS, incubated at 62°C for 10 min and quenched with 50 μl 10% Triton X-100 and 290 μl water at 37°C for 15 min. Digestion was performed overnight by adding 50 μl HindIII (New England Biolabs) buffer and 10 μl high-concentration HindIII and incubating the samples at 37°C in a thermomixer. Before this step, an aliquot was taken from each sample as an undigested control. Digests were heat inactivated for 20 min at 65°C and an aliquot was taken from each sample as a digested (unligated) control. Samples were cooled at room temperature for 10 min before adding the ligation cocktail. 3C libraries were ligated for 4 h at 25°C with 10U T4 ligase and ligation buffer (Thermo Fisher Scientific) in a thermomixer at 100 ***g***. Ligated samples were then centrifuged at 300 ***g***, resuspended in 240 µl 5% SDS and 1 mg Proteinase K, incubated at 55°C for 30 min, supplemented with 50 µl 5 M NaCl and incubated at 65°C for 4 h. DNA was then purified by adding 500 µl isopropanol, incubated at −80°C overnight, centrifuged at 13,000 ***g*** at 4°C, washed with 70% EtOH, air dried and resuspended in 100 µl water, followed by incubation with RNase A at 37°C for 1 h. 3C templates were quantified using Qubit DNA Broad-Range (Thermo Fisher Scientific) and diluted to 100 ng/µl. Libraries and respective controls (undigested and digested aliquots) were verified on a gel.

5C was performed using the method described by Nora et al. (2017), which adopts a single-PCR strategy to construct 5C-sequencing libraries from the 3C template. Briefly, four 10 µl 5C annealing reactions were assembled in parallel, each using 500 ng of 3C template, 1 µg salmon sperm (Thermo Fisher Scientific) and 10 fmol of each 5C oligonucleotide in 1× NEBuffer 4 [5C set of oligonucleotides described by Nora et al. (2012)]. Samples were denatured at 95°C for 5 min and incubated at 48°C for 16-18 h. Then, 10 µl of 1× Taq ligase buffer with 5U Taq ligase was added to each annealing reaction followed by incubation at 48°C for 4 h and 65°C for 10 min. Negative controls (no ligase, no template or no 5C oligonucleotide) were included during each experiment to ensure the absence of contamination. To attach Illumina-compatible sequences, 5C libraries were directly PCR amplified with primers harboring 50-mer tails containing Illumina sequences that anneal to the universal T3/T7 portion of the 5C oligonucleotides (Nora et al., 2017). For this, each 5C ligation reaction was used as the template for three parallel PCRs (12 PCRs in total), using 6 µl 5C ligation with 1.125 U AmpliTaq Gold (Thermo Fisher Scientific) per reaction in 1× PCR Buffer II, 1.8 mM MgCl_2_, 0.2 mM dNTPs and 1.25 mM primers in a total of 25 ml. Cycling conditions were: 95°C for 9 min, 25 cycles of 95°C for 30 s, 60°C for 30 s, 72°C for 30 s followed by 72°C for 8 min. PCR products from the same 3C sample were pooled and run on a 2.0% agarose electrophoresis gel. 5C libraries (231 bp) were then excised and purified with the MinElute Gel Extraction kit (QIAGEN). Library concentrations were estimated using TapeStation (Agilent) and Qubit (Thermo Fisher Scientific), pooled and sequenced using 12 pM for loading on rapid flow cells using the HiSeq 2500 system (Illumina). Sequencing mode was set as 20 dark cycles followed by 80 bases in single-end reads (SR80).

Sequencing data were processed using our custom pipeline, 5C-Pro, available at https://github.com/bioinfo-pf-curie/5C-Pro. Briefly, single-end sequencing reads were first trimmed to remove Illumina adapters and aligned on an *in silico* reference of all pairs of forward and reverse primers using the bowtie2 software (Langmead and Salzberg, 2012). Aligned reads were then directly used to infer the number of contacts between pairs of forward and reverse primers, thus providing a 5C map at primer resolution. Based on our previous experiments, inefficient primers were discarded from the downstream analysis. Quality controls of the experiments were then performed using the HiTC BioConductor package (Servant et al., 2012). Data from biological replicates were pooled (summed) and binned using a running median (window=30 kb, final resolution=6 kb). We normalized 5C contacts for the total number of reads and filtered out outlier probes and singletons, as previously described (Hnisz et al., 2016; Nora et al., 2012; Smith et al., 2016). We also developed a novel method to exclude noisy contacts in the 5C maps, called ‘neighborhood coefficient of variation’, available at https://github.com/zhanyinx/Coefficient_Variation. Considering that the chromatin fiber behaves as a polymer, the contact frequency of a given pair of genomic loci (e.g. *i* and *j*) cannot be very different from that of fragments *i*±*N* and *j*±*N* if *N* is smaller (or in the order of) than the persistence length of the chromatin fiber. Hence, a given pixel in the 5C map (which is proportional to the contact frequency between the two corresponding loci) can be defined as noisy if its numerical value is too different from those corresponding to neighboring interaction frequencies. To assess the similarity of a given interaction with neighboring contacts operatively, we calculated the coefficient of variation (CV) of contacts (pixels in the 5C map) in a 10×10 square centered on every contact. We then set out to discard pixels for which the corresponding CV was above a threshold. Given that the distribution of the CV of all 5C samples in this study was bimodal around CV=1, we set the CV threshold to 1. Discarded contacts appeared as gray pixels in the differential 5C maps. For differential analysis between two samples of interest, we calculated the difference between *z*-scores determined for each individual map (Smith et al., 2016). Samples corresponding to inversions of genomic regions were mapped to a virtually inverted map before analysis. Samples corresponding to deletions were corrected for the new distance between genomic elements; this distance adjustment was performed along with the *z*-score calculation. 5C data for the E14 cell line (used as control) have been published previously (Galupa et al., 2020) but control and mutant samples were collected and processed in parallel.

### Statistical analysis

For RNA FISH, nCounter and allelic expression analysis, details of the statistical analyses used are provided in the figure legends, figures and/or Results, including the statistical tests used, exact value of *n* and what *n* represents.

## Supplementary Material

Supplementary information

Reviewer comments
