# Supplementary material for: Inversion of a topological domain leads to restricted changes in its gene expression and affects interdomain communication
Source: Development. 2022 May 6;149(9):dev200568. doi: 10.1242/dev.200568 (PMC9148567; doi:10.1242/dev.200568)
Supplement: Supplementary information [file develop-149-200568-s1.pdf]

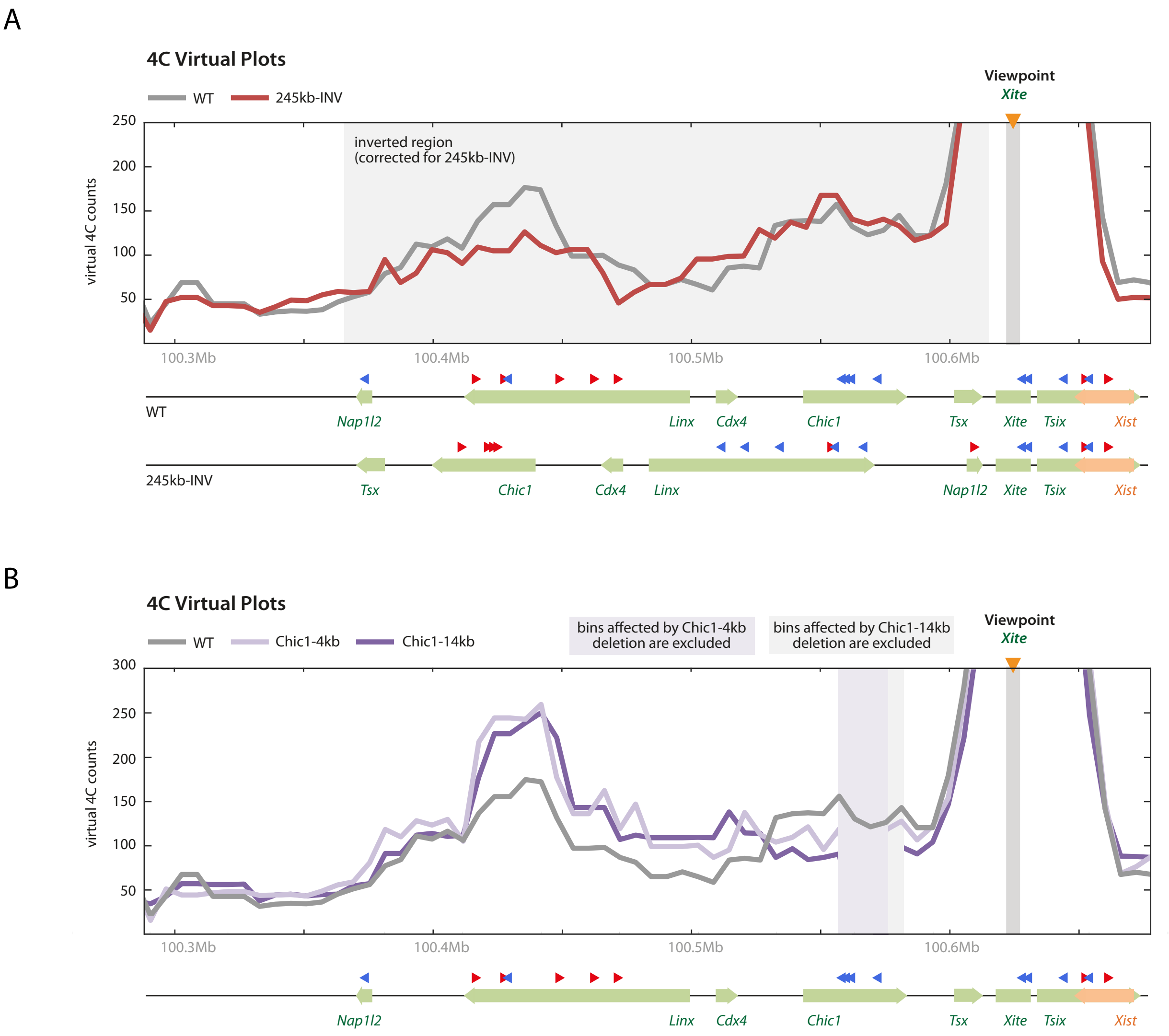


**Fig. S1.** (*A*) Virtual 4C plots for the wild type (WT) and 245kb-INV alleles, for which the anchor is the element *Xite*. The interaction frequency between *Xite-Tsx* is ~5-fold lower in the inverted allele (~50 counts) than in the WT (~250 counts, and within the region where contact frequency is dominated by genomic distance). Inversely, the interaction frequency between *Xite-Nap1L2* in the inverted allele (~250 counts) is ~5-fold higher than in the WT (~50 counts). The changes in interaction frequencies between these elements seem thus to reflect the changes in genomic distances for WT and inverted alleles. (*A*) Virtual 4C plots for the wild type (WT) and deletion alleles Chic1-4kbD and Chic1-14kbD. The interaction frequency between *Xite* and *Linx* is increased in the mutant alleles compared to WT.

Development • Supplementary information

# Figure S2

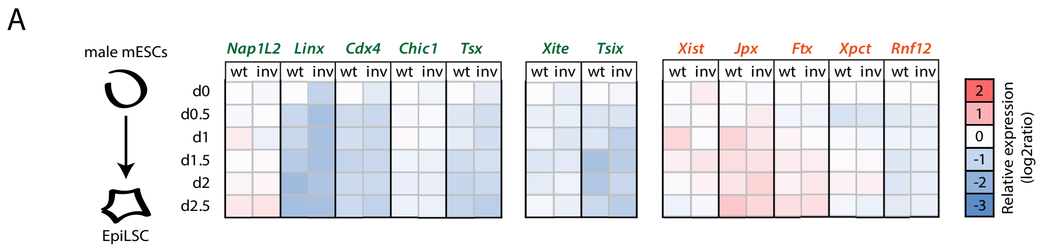


**Fig. S2.** (A) Heatmap representation of Nanostring nCounter analysis of wild-type (wt) and Linx-51kb-INV (inv) mESCs during differentiation. Data for each gene is normalised to wt-d0, and represents the average of two biological replicates (wt) or of two independent clones (inv).
