# Supplementary material for: Inversion of a topological domain leads to restricted changes in its gene expression and affects interdomain communication
Source: Development. 2022 May 6;149(9):dev200568. doi: 10.1242/dev.200568 (PMC9148567; doi:10.1242/dev.200568)
Supplement: Reviewer comments [file develop-149-200568_review_history.pdf]

Inversion of a topological domain leads to restricted changes in its gene expression and affects interdomain communication

Rafael Galupa, Christel Picard, Nicolas Servant, Elphège P. Nora, Yinxiu Zhan, Joke G. van Bemmel, Fatima El Marjou, Colin Johanneau, Maud Borensztein, Katia Ancelin, Luca Giorgetti and Edith Heard
DOI: 10.1242/dev.200568

Editor: James Briscoe

Review timeline
Submission to Review Commons: 7 August 2021
Original submission: 21 January 2022
Editorial decision: 28 February 2022

Reviewer 1

Evidence, reproducibility and clarity

Galupa et al. use a large inversion inside the Tsix TAD to investigate the role of chromatin contact rearrangements within TADs on the regulation of this locus. The results are largely consistent with current models of gene regulation by distal regulatory elements, and are generally well supported by the figures and data shown. Some aspects could use some additional support to rule out alternative explanations for the results, and a few aspects of the figures were unclear.

Major comments

1.The authors claim that the changes in expression of Nap1 L2 and Tsx are due to changes in their genomic distance from the Xite element. However, interaction frequency generally correlates with genomic distance, and they do not explore whether interaction frequency between these regions is affected by the inversion. It is difficult to identify the strengths of interactions involving these regions from the visualisations provided. In order to support this claim, the authors could provide a quantification of the interaction strengths between the Xite element and Nap1 L2/Tsx in the WT and inverted alleles. This could be done with a "virtual 4C" track or by quantifying average interactions in a window around the elements.

2.The authors also claim that the regulation of Tsix by hypothetical regulatory sequences within the inverted region depends on the orientation of the sequences. While they show that regulation of Tsix depends on the orientation of the regions as a whole, this phrasing implies that the orientation of individual regulatory elements is also important, which is not supported by the data shown. This should be rephrased for clarity, or additional data shown to support this point.

Minor comments

- Figure 2A: it is unclear where the Tsix/Xist TADs are relative to the larger region shown here, as there is no scale or annotations. Similarly, the region shown in 2D does not appear to directly correspond to the region shown in the other panels. The authors should either add genomic coordinates to the figures, add gene annotations, or annotate the TADs as done in other figures, so that the reader can understand how they relate to each other.

- 1 was confused by Figure 2B initially as the orientation of the Xite CTCF sites is not clear. Given the density of CTCF sites, the colour is a more useful marker of orientation than the arrowhead, so

© 2022. Published by The Company of Biologists under the terms of the Creative Commons Attribution License (https://creativecommons.org/licenses/by/4.0/).

it would make sense to swap the colour of the inverted sites to match the new orientation rather than the original, as has been done in Figure 1 B.

- Is there anything notable about the region the Linx cluster forms new interactions with in the inverted allele? Does it have CTCF binding or enhancer signatures that could potentially contribute to some of the observed gene expression changes?

Significance

This study uses a large inversion inside the Tsix TAD to investigate the role of chromatin contact rearrangements within TADs on the regulation of this locus. The results are largely consistent with current models of gene regulation by distal regulatory elements, and are generally well supported by the figures and data shown.

This study will be of interest to chromatin biology researchers as an additional study that reflects the complex interrelation between chromatin organisation and regulation of gene expression.

Field of expertise: chromatin organisation; regulation of gene expression.

Referees cross commenting
I (Reviewer #1) also largely agree with the points raised by the other reviewers, but some of the revisions might be out of scope of the manuscript. In particular:

-Reviewer #2 suggests either rewriting part of the discussion referring to a possible role of Tsix downregulation in Xist upregulation, or doing RNA-FISH or scRNA-seq experiments to further investigate this. In my opinion, rephrasing the discussion would be sufficient here.

-Reviewer #2 also suggests 4C experiments but, in my opinion, virtual 4C would work well for visualising interactions of specific viewpoints.

-Reviewer #3 noted the sex ratio skew in Figure 4 - I also noticed this before but assumed it wasn't significant since the authors didn't discuss it. The proposed experiments by Reviewer #3 would strengthen the manuscript, but they seem out of scope. The authors could comment on this in their discussion.

Reviewer 2

Evidence, reproducibility and clarity

Galupa et al. tested how inversing of the internal part of topologically associated domain (TAD) within murine X inactivation center (XIC) would affect local chromatin interactions and gene expression. XIC locus is crucial for the initiation of X-chromosome inactivation in mammalian female cells and harbors long noncoding RNA Xist and other regulatory elements necessary for transcriptional shutdown of one of X chromosomes. XIC is organized into two TADs: one harboring Xist promoter along with positive regulators of Xist ("Xist-TAD") and the second, adjacent TAD harboring of Tsix coding for the Xist antisense transcript blocking its upregulation as well as other repressors.

Authors inverted most of the domains within the Tsix-TAD interior (-80%) with the aid of CRISR/Cas9 genome engineering in male mESC while leaving the boundaries intact. The changes in the interactions were investigated with carbon copy chromosome conformation capture (5C) allowing high resolution detection of chromatin contacts within defined genomic region combined with CTCF ChIP-seq analysis depicting potential insulation points. Similarly, to wild-type cells (WT) in inversion mutant (INV) three interaction hot spots could be observed within Tsix-TAD and they involving the same loci Linx and Chic1 from the inverted part of the TAD and Tsix enhancer Xite from the 3' part of the TAD which was outside of the inversion.
Interestingly, this led to interactions pattern resembling those in WT cells. Authors convincingly explain this with the fact that due to the structure of the locus and the design of the inversion the distribution and directions of CTCF sites within Xist-TAD in INV cells were similar. Chic1 interaction

© 2022. Published by The Company of Biologists under the terms of the Creative Commons Attribution License (https://creativecommons.org/licenses/by/4.0/).

hot spot took over Linx locus "structural" role and both were after inversion in similar configuration to the WT in relation to not moved Xite-Tsix end of the TAD. At the same time those "structurally equivalent" interactions were obviously new. Besides striking similarities there were also significant differences: increased long-range interactions involving Linx upstream of inverted region as well as reduced interactions with Chic1. Those differences likely reflect differences in potential of CTCF from both loci to form chromatin contacts. Furthermore, with insulation analysis authors showed that in INV cells insulation between Tsxi-TAD and Xist-TAD is stronger.

Observed changes in Tsix-TAD were associated with downregulation of Tsix and additionally affected expression of two genes at the end of inverted region: downregulated Tsx and highly upregulated Nap1 L2 suggesting likely effect of promoter-enhancer rewiring in the INV cells. Authors performed also expression analysis during mESCs differentiation and found out that Tsix downregulation was not maintained. Expression of other genes did not show differences. Interestingly authors notice upregulation of Xist during differentiation and after further investigation with RNA-FISH they conclude that it is caused by the Xist expression in 4-7% of used mESCs seen as Xist clouds typically seen in female cells.
Analysis of gene expression in the heterozygous mouse harboring similar inversion within Tsix-TAD as in studied male mESCs showed convincingly moderately increased Xist level with now differences in the frequency of gene silencing of genes originating from either WT or INV alleles.

Overall data generated by Galupa et al. presented in the "Results" section of this study are presented in a clear, convincing way and support their conclusions. Some minor comments and suggestions could be found below.
Discussion is thorough, well written and reviewer agrees with almost all statements apart form one: he doesn't share the confidence that Xist upregulation can't relate to Tsix downregulation. Suggested explanation that Xist upregulation may be due to the Tsx downregulation is believable. Authors argue that Tsix downregulation happens only in pluripotent state not during differentiation when Xist gets upregulated. Shown Tsix downregulation is relatively small and it raises the question if it is due to small downregulation in most cells or stronger/strong downregulation in part of the cells. Xist clouds were found in the 4-7% of the INV cells, possibly in those cells there was stronger downregulation of Tsix compared to other without Xist clouds. In reviewer's opinion one could not exclude the role of Tsix in Xist upregulation without extra experiments like RNA-FISH showing whether expression of Tsix has changed in cells with Xist clouds.

Major comments:
-In the discussion part regarding lack of the role of Tsix downregulation in the Xist upregulation should be rewritten unless additional RNA-FISH or single cell RNA-seq experiments are done to support this statement

Minor comments:

-It should be stressed in the first paragraph of the paper as well as in the figure 1 and figure description that the boundaries of Tsix-TAD were not affected during (In the first part of the manuscript it is mentioned only in the abstract). In the figure 1B it would be helpful for the reader to mark the 5' Tsix TAD border also insulation plot (as in Fig. 2D, but aligned below Fig. 1A).

-Referring to the new structurally similar interactions as "eqivalent" is a bit misleading reviewer thinks that "structurally similar" would be better

-In Fig. 2A no genomic coordinates are provided neither on the figure not in the legend, also which region of Fig. 2A is zoomed in in Fig.2C should be marked. Whenever possible genomic coordinates should be given.

-In the manuscript text it should have been mentioned that presented 5C heatmaps for INV cells are "inversion corrected" as stated in the legend, possibly with short explanation what does it mean - i.e. coordinates for 5C INV heatmap were reshuffled to reflect new structure of Tsix-TAD in the INV cells. Not "inversion corrected" heatmap should be provided in supplemental figure
-Gene in Fig. 3B Huwe1 is not referred to in any way in the manuscript. It would be good to add at least in the legend int coordinates or information that it is located outside of XIC. If it was included to investigate potential although rather unlikely Xist silencing some quantification could be added

© 2022. Published by The Company of Biologists under the terms of the Creative Commons Attribution License (https://creativecommons.org/licenses/by/4.0/).

-It would be beneficial for the study if additional RNA FISH experiments studying single cell expression changes could be done particularily for Tsix. Observed changes are small and possibly bulk analysis masks bigger changes in part of single cells.

-it may be beneficial for the research community to publish the study at cureent stage, however adding additional epxperiments like 4C with view point localized within interaction hubs woudl give much detiled insight into rewiring of interactions

Significance

Nature and significance of the advance, comparison to existing knowledge.

Findings of Galupa et al. provide new valuable insight on the highly disputed topic of the role of 3D chromatin organization in gene expression control.

Currently it is still poorly understand to what extent structures like TADs regulate gene expression. As overviewed nicely in the manuscript introduction, experiments of other groups showed that for some cells removing TADs affects expression of surprisingly small number genes on the other hand there are many examples where distorting TADs caused large changes in local gene expression. This suggests that the effects depend on the context of 1.) developmental biology - time restricted "developmental windows" crucial for establishment of proper expression programmes and 2.) genetic context - some TADs or genes depend more on the 3D chromatin organization as their regulator.

Those contexts are so far poorly understood and studies like Galupa et al. increase this understanding providing new examples and mechanistic insights of how genes at distinct loci are being regulated. Reviewed study showed for the first time that set of genes within the murine Xist-TAD depends to different extent on the local chromatin structure and provided insightful convincing suggestions of why this happens.

Apart form significance to broard audience interested in gene regulation this study advances also understanding of how X inactivation - process crucially important for mammals is regulated. In particular Authors add important insights of how Tsix, Xist regulator, is controlled extending their findings from the previous publications.

Audience:

Broad audience interested in the role of the chromatin folding on the gene expression in general, also X inactivation filed interested in its regulation

Reviewers expertise:

Epigenetic regulation of gene expression, 3D genome, X inactivation, developmental biology, bioinformatics

Referees cross commenting

I (Reviewer #2) agree largely with the comments of Reviewer #1 & #3. The additional experiments suggested by Reviewer #3 would strengthten the manuscript, but in my view this would mean a major revision and I am not conviced they should be mandatory.

Reviewer 3

Evidence, reproducibility and clarity

This study investigates phenotypes resulting from a TAD inversion at the X-inactivation center (Xic). The authors generated a 245kb inversion of the Tsix-TAD, which includes various CTCF clusters, and excludes the Xite and Tsix genes. The authors introduced this inversion into male embryonic stem cells (ESCs) and also made a transgenic mouse with this construct. This

© 2022. Published by The Company of Biologists under the terms of the Creative Commons Attribution License (https://creativecommons.org/licenses/by/4.0/).

manuscript is well-written, and has some exciting data that will advance the field of nuclear organization and X-chromosome Inactivation. Comments and suggestions are listed below:

1.1t is unclear why the authors excluded the Xite gene from the Tsix-TAD inversion. Is there a reason why Xite and its associated CTCF sites remained intact? Given that Xite functions as a transcriptional regulator of Xist, maintaining the original site of Xite (which is part of the Tsix TAD) may/may not influence the results.

2.In Figure 4, the authors demonstrate that Xist expression was increased in F1 female transgenic embryos, yet XCI was not skewed. In Figure 4D, when the inversion is inherited from the maternal allele, there was a small yet possibly significant reduction in female embryos (19 WT vs 9). In addition, the litter size seems to be reduced. These data suggest that the inversion may have more phenotypic consequences. Perhaps additional litters should be genotyped, and embryos harvested earlier to determine if there are increased gene expression changes prior to embryo reabsorption. Because XCI is female-specific, it would be best if the inversion experiments were performed in female ESCs instead of male cells, or the phenotypes resulting from inversion should be investigated in more depth in female embryos.

3.This study found that the Tsix-TAD inversion leads to upregulated Xist expression in male ESCs and female F1 embryos. The authors suggest that the cause of Xist upregulation could be the result of altered orientation of the CTCF sites within the TAD inversion. However, the authors did not directly test this hypothesis. One suggestion would be to investigate which CTCF sites are responsible for Xist upregulation, by performing TAD inversions on smaller regions of the Tsix-TAD. Using a loss of function, and also perhaps gain of function approaches for specific CTCF sites within this Tsix-TAD would reveal a hierarchy of TAD strength within this region of the Xic, and may also result in larger gene expression changes.

Significance

This study investigates how TAD inversion (genome topology) impacts gene expression and also female development. THere have been numerous studies that mutate CTCF binding or TAD organization, and frequently there are no significant gene expression changes. This study found that inverting a TAD within the X-inactivation Center impacted Xist upregulation and may also have a phenotype in female embryos, which could be explored further.

This study will be of interest to the fields of nuclear organization and X-chromosome Inactivation.

<u>Author response to reviewers' comments</u>

Point-by-point Letter to Reviewer's Comments

<u>Reviewer #1 (Evidence, reproducibility and clarity (Required)):</u>
Galupa et al. use a large inversion inside the Tsix TAD to investigate the role of chromatin contact rearrangements within TADs on the regulation of this locus. The results are largely consistent with current models of gene regulation by distal regulatory elements, and are generally well supported by the figures and data shown. Some aspects could use some additional support to rule out alternative explanations for the results, and a few aspects of the figures were unclear.

We thank the reviewer for their insightful analysis of our manuscript and for the helpful comments.

Major comments
1. The authors claim that the changes in expression of Nap1L2 and Tsx are due to changes in their genomic distance from the Xite element. However, interaction frequency generally correlates with genomic distance, and they do not explore whether interaction frequency between these regions is affected by the inversion. It is difficult to identify the strengths of interactions involving these regions from the visualisations provided. In order to support this claim, the authors could provide a quantification of the interaction strengths between the Xite element and Nap1L2/Tsx in the WT

© 2022. Published by The Company of Biologists under the terms of the Creative Commons Attribution License (https://creativecommons.org/licenses/by/4.0/).

and inverted alleles. This could be done with a "virtual 4C" track or by quantifying average interactions in a window around the elements.

Answer #1: We thank the reviewer for this suggestion. We have now generated virtual 4C plots for the WT and inverted alleles (see below; also included as Fig. S1 in the manuscript), for which the anchor is the element *Xite*. As observed in the plots, the interaction frequency between *Xite-Tsx* is ~5-fold lower in the inverted allele (~50 counts) than in the WT (~250 counts, and within the region where contact frequency is dominated by genomic distance). Inversely, the interaction frequency between *Xite-Nap1L2* in the inverted allele (~250 counts) is ~5-fold higher than in the WT (~50 counts). The changes in interaction frequencies between these elements seem thus to reflect the changes in genomic distances for WT and inverted alleles.

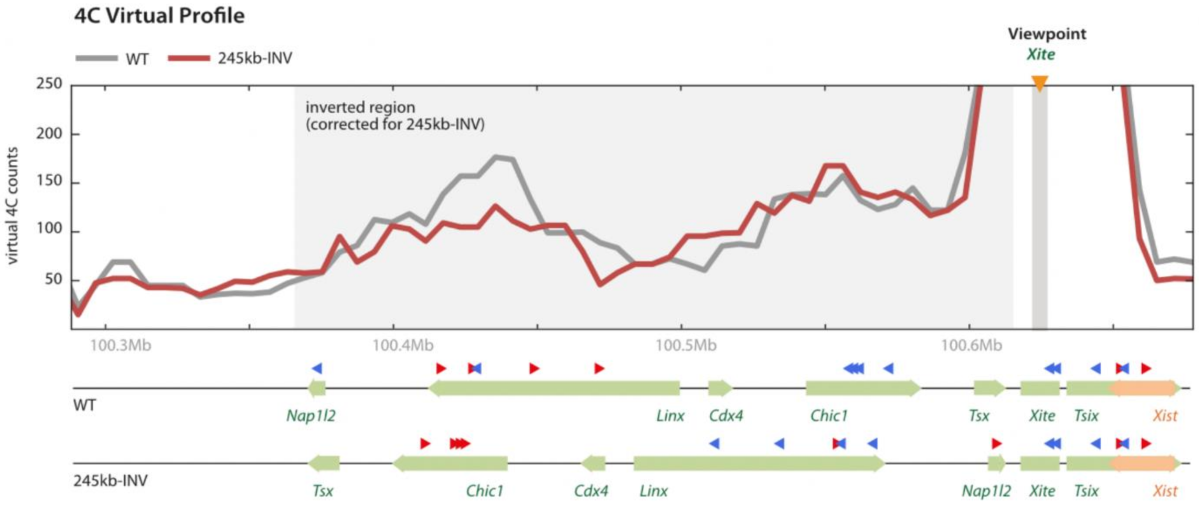

Accordingly, we added these explanations to the figure legend, and added the underlined sentence to the manuscript:

"Since deletion of *Xite* leads to downregulation of *Tsx* (van Bemmel et al., 2019), moving *Tsx* away from *Xite* on the 245kb-INV allele could lead to its observed downregulation. Conversely, increased linear proximity of *Nap1L2* to *Xite* could possibly underlie *Nap1L2* upregulation. Changes in interaction frequencies between *Xite* and these two elements in the 245kb-INV allele do support this hypothesis, as they reflect the changes in their genomic distances (increased for *Xite-Nap1L2* and decreased for *Xite-Tsx*, compared with control, **Fig. S1**)."

2. The authors also claim that the regulation of Tsix by hypothetical regulatory sequences within the inverted region depends on the orientation of the sequences. While they show that regulation of Tsix depends on the orientation of the regions as a whole, this phrasing implies that the orientation of individual regulatory elements is also important, which is not supported by the data shown. This should be rephrased for clarity, or additional data shown to support this point.

Answer #2: We thank the reviewer for pointing this out. We have rephrased the sentence for clarity, which now reads as:

"together with the current data, this suggests that the region contains important sequences for Tsix regulation and that this regulation depends on the orientation of the region as a whole, and might depend on the orientation of individual regulatory sequences."

Minor comments

•Figure 2A: it is unclear where the Tsix/Xist TADs are relative to the larger region shown here, as there is no scale or annotations. Similarly, the region shown in 2D does not appear to directly correspond to the region shown in the other panels. The authors should either add genomic coordinates to the figures, add gene annotations, or annotate the TADs as done in other figures, so that the reader can understand how they relate to each other.

© 2022. Published by The Company of Biologists under the terms of the Creative Commons Attribution License (https://creativecommons.org/licenses/by/4.0/).

Answer #3: We apologise for this lack of clarity in the figures, indeed they can benefit from additional information. We have now added genomic coordinates for Fig. 2A, as well as annotations of the Tsix- and Xist-TADs. For Fig. 2D, we have added annotations for the Tsix- and Xist-TADs as done in other figures (and genomic coordinates were already present). We thank the reviewer for noting these omissions.

•I was confused by Figure 2B initially as the orientation of the Xite CTCF sites is not clear. Given the density of CTCF sites, the colour is a more useful marker of orientation than the arrowhead, so it would make sense to swap the colour of the inverted sites to match the new orientation rather than the original, as has been done in Figure 1B.

Answer #4: This is a good point; we changed the colours accordingly and thank the reviewer for pointing this out.

•Is there anything notable about the region the Linx cluster forms new interactions with in the inverted allele? Does it have CTCF binding or enhancer signatures that could potentially contribute to some of the observed gene expression changes?

Answer #5: It is a very good question; we have investigated this previously but found no remarkable signatures for this region (either CTCF or active chromatin marks). We have added one phrase to state this:

"Increased contacts could be observed upstream of the inverted region, corresponding to contacts stemming from the Linx CTCF cluster in its new position (Fig. 2B, bottom, black arrow, red region in the differential map; this region shows no particular chromatin signatures, such as CTCF binding or active chromatin marks)."

Referees cross commenting

I (Reviewer #1) also largely agree with the points raised by the other reviewers, but some of the revisions might be out of scope of the manuscript.

We warmly thank the reviewer for commenting the feedback from other reviewers.

In particular:

-Reviewer #2 suggests either rewriting part of the discussion referring to a possible role of Tsix downregulation in Xist upregulation, or doing RNA-FISH or scRNA-seq experiments to further investigate this. In my opinion, rephrasing the discussion would be sufficient here.

We thank the reviewer for this comment. We have rephrased the discussion accordingly, please see Answer #6.

-Reviewer #2 also suggests 4C experiments but, in my opinion, virtual 4C would work well for visualising interactions of specific viewpoints.

We thank the reviewer for this comment. We have added the virtual 4C profiles to the manuscript as suggested by Reviewer #1 themselves, please see Answer #1.

-Reviewer #3 noted the sex ratio skew in Figure 4 - I also noticed this before but assumed it wasn't significant since the authors didn't discuss it. The proposed experiments by Reviewer #3 would strengthen the manuscript, but they seem out of scope. The authors could comment on this in their discussion.

We thank the reviewer for this comment. Please see Answer #15 to Reviewer #3.

Reviewer #2 (Evidence, reproducibility and clarity (Required)):
Overall data generated by Galupa et al. presented in the "Results" section of this study are presented in a clear, convincing way and support their conclusions. Some minor comments and suggestions could be found below.

© 2022. Published by The Company of Biologists under the terms of the Creative Commons Attribution License (https://creativecommons.org/licenses/by/4.0/).

Discussion is thorough, well written and reviewer agrees with almost all statements apart form one: he doesn't share the confidence that Xist upregulation can't relate to Tsix downregulation. Suggested explanation that Xist upregulation may be due to the Tsx downregulation is believable. Authors argue that Tsix downregulation happens only in pluripotent state not during differentiation when Xist gets upregulated. Shown Tsix downregulation is relatively small and it raises the question if it is due to small downregulation in most cells or stronger/strong downregulation in part of the cells. Xist clouds were found in the 4-7% of the INV cells, possibly in those cells there was stronger downregulation of Tsix compared to other without Xist clouds. In reviewer's opinion one could not exclude the role of Tsix in Xist upregulation without extra experiments like RNA-FISH showing whether expression of Tsix has changed in cells with Xist clouds.

We thank the reviewer for his encouraging comments and thorough analysis of the manuscript.

Major comments:

-In the discussion part regarding lack of the role of Tsix downregulation in the Xist upregulation should be rewritten unless additional RNA-FISH or single cell RNA-seq experiments are done to support this statement

Answer #6: We thank the reviewer for this comment – we have rewritten the discussion to better reflect that we cannot exclude that *Xist* upregulation is related to *Tsix* downregulation. It now reads as:
"This upregulation could be associated with one or more of the other alterations observed on the 245kb-INV allele, either structural, or transcriptional, or both. For instance, we observed reduced expression for Tsix, Xist's antisense cis-repressor, in the pluripotent state, which could have an impact in Xist regulation; during differentiation, however, when Xist is upregulated, we did not detect differences in Tsix expression. Further research will be needed to clarify the involvement of Tsix in the Xist phenotype observed here."

We also removed a passage (underlined) in the discussion that read as "Downregulation of Tsx in 245kb-INV mutant cells might thus account, partially or maybe even completely, to ectopic Xist upregulation – but independently of Tsix or Xite expression."

Minor comments:

-It should be stressed in the first paragraph of the paper as well as in the figure 1 and figure description that the boundaries of Tsix-TAD were not affected during (In the first part of the manuscript it is mentioned only in the abstract). In the figure 1B it would be helpful for the reader to mark the 5' Tsix TAD border also insulation plot (as in Fig. 2D, but aligned below Fig. 1A).

Answer #7: We thank the reviewer for these good points.

Regarding the text, we have added information accordingly as follows (underlined):

"Using a CRISPR/Cas9 editing approach in male mESCs, which carry a single X chromosome, we targeted a ~245kb region encompassing all loci within the Tsix-TAD, including the CTCF clusters within Linx and Chic1, but excluding Xite and Tsix (Fig. 1B). The targeted region does not involve either of the two boundaries of the TAD. We successfully generated two clones harbouring an inversion allele (245kb-INV) (Fig. 1C)."

We have also added this information to the figure description: "(B) Targeting strategy for inverting the ~245kb region comprising most of the Tsix-TAD, except Tsix and its known regulator Xite, and leaving the boundaries intact."
Regarding the figure, and as requested by the reviewer, we have included the corresponding insulation plot below Fig. 1A.

-Referring to the new structurally similar interactions as "equivalent" is a bit misleading reviewer thinks that "structurally similar" would be better
Answer #8: We changed the text according to the reviewer's suggestion.

© 2022. Published by The Company of Biologists under the terms of the Creative Commons Attribution License (https://creativecommons.org/licenses/by/4.0/).

-In Fig. 2A no genomic coordinates are provided neither on the figure not in the legend, also which region of Fig. 2A is zoomed in in Fig.2C should be marked. Whenever possible genomic coordinates should be given.

Answer #9: We thank the reviewer for these suggestions. We have added genomic coordinates to Fig. 2A and included reference points in Fig. 2C. Please see as well Answer #3 to Reviewer #1.

-In the manuscript text it should have been mentioned that presented 5C heatmaps for INV cells are "inversion corrected" as stated in the legend, possibly with short explanation what does it mean - i.e. coordinates for 5C INV heatmap were reshuffled to reflect new structure of Tsix-TAD in the INV cells. Not "inversion corrected" heatmap should be provided in supplemental figure

Answer #10: We thank the reviewer for this suggestion, which we have implemented in the manuscript text. We will also include the "non-corrected" map as supplementary figure.

-Gene in Fig. 3B Huwe1 is not referred to in any way in the manuscript. It would be good to add at least in the legend int coordinates or information that it is located outside of XIC. If it was included to investigate potential although rather unlikely Xist silencing some quantification could be added.

Answer #11: We have added now this information to the figure legend according to the reviewer's suggestion: "(C) RNA FISH for Huwe1 (X-linked gene outside of the Xic) (…)"
The Methods section contains the reference for the BAC used as a probe to detect *Huwe1*, which allows one to identify its coordinates. We chose to detect *Huwe1* as a way to "mark" the X-chromosome and as a positive control for the detection efficiency of the FISH.

-It would be beneficial for the study if additional RNA FISH experiments studying single cell expression changes could be done particularily for Tsix. Observed changes are small and possibly bulk analysis masks bigger changes in part of single cells.

Answer #12: We agree with the reviewer that it would be interesting to further investigate the relationship between *Xist* and *Tsix* expression at the single-cell level; however, in our experience, quantitative RNA FISH is not a trivial experiment (especially for small effects). We have changed the text according to the reviewer's suggestion regarding the relationship between *Xist* and *Tsix* expression in these mutants (please see Answer #6), also in light of Reviewer #1's cross-comments.

-it may be beneficial for the research community to publish the study at cureent stage, however adding additional epxperiments like 4C with view point localized within interaction hubs woudl give much detiled insight into rewiring of interactions

Answer #13: We thank the reviewer for this comment. We have now added virtual 4C profiles (based on the 5C data) for better inspection of interaction between specific viewpoints; please see Answer #1.

Referees cross commenting
I (Reviewer #2) agree largely with the comments of Reviewer #1 & #3. The additional experiments suggested by Reviewer #3 would strengthten the manuscript, but in my view this would mean a major revision and I am not conviced they should be mandatory.

We warmly thank the reviewer for providing feedback on the other reviewers' comments.

Reviewer #3 (Evidence, reproducibility and clarity (Required)):

This study investigates phenotypes resulting from a TAD inversion at the X-inactivation center (Xic). The authors generated a 245kb inversion of the Tsix-TAD, which includes various CTCF clusters, and excludes the Xite and Tsix genes. The authors introduced this inversion into male embryonic stem cells (ESCs) and also made a transgenic mouse with this construct. This manuscript is well-written, and has some exciting data that will advance the field of nuclear organization and X-chromosome Inactivation.

© 2022. Published by The Company of Biologists under the terms of the Creative Commons Attribution License (https://creativecommons.org/licenses/by/4.0/).

We thank the reviewer for their thorough analysis of our manuscript and for their encouraging comments.

Comments and suggestions are listed below:
1. It is unclear why the authors excluded the Xite gene from the Tsix-TAD inversion. Is there a reason why Xite and its associated CTCF sites remained intact? Given that Xite functions as a transcriptional regulator of Xist, maintaining the original site of Xite (which is part of the Tsix TAD) may/may not influence the results.

Answer #14: We thank the reviewer for raising this point. Based on the results presented in our manuscript, we believe that including or not including *Xite* in the inversion would indeed influence the results. We thought that not touching *Xite* would be more informative for two reasons: (i) *Xite* is already known to influence *Xist* expression (via *Tsix*); and (ii) if *Xite* was inverted along with the rest of the TAD, the relative CTCF orientations between *Xite*/*Linx*/*Chic1* would not have changed, and we wanted to "induce" topological alterations. For clarity, we now have included these explanations in the manuscript text, as underlined below:

"Using a CRISPR/Cas9 editing approach in male mESCs, which carry a single X chromosome, we targeted a ~245kb region encompassing all loci within the Tsix-TAD, including the CTCF clusters within *Linx* and *Chic1*, but excluding *Xite* and *Tsix* (**Fig. 1B**). We decided not to include *Xite* in the inversion because (i) *Xite* is already known to influence *Xist* expression (via *Tsix*), and (ii) if *Xite* was inverted along with the rest of the TAD, the relative CTCF orientations between *Xite*, *Linx* and *Chic1* would not have changed."

2. In Figure 4, the authors demonstrate that Xist expression was increased in F1 female transgenic embryos, yet XCI was not skewed. In Figure 4D, when the inversion is inherited from the maternal allele, there was a small yet possibly significant reduction in female embryos (19 WT vs 9). In addition, the litter size seems to be reduced. These data suggest that the inversion may have more phenotypic consequences. Perhaps additional litters should be genotyped, and embryos harvested earlier to determine if there are increased gene expression changes prior to embryo reabsorption. Because XCI is female-specific, it would be best if the inversion experiments were performed in female ESCs instead of male cells, or the phenotypes resulting from inversion should be investigated in more depth in female embryos.

Answer #15: We thank the reviewer for bringing up these points. Litter size is indeed reduced, which might indeed suggest that the inversion may have more phenotypic consequences; we added a sentence (underlined) to the manuscript text to highlight this observation:

"Thus, the 245kb inversion leads to higher *Xist* levels in *cis* but this does not result in skewed patterns of X- inactivation. Of note, litter size seems to be reduced upon maternal transmission of the 245kb-INV allele, with a skewed sex ratio (71% females in 245kb-INV, 59% in control) suggesting that the inversion may have more phenotypic consequences."

There is no reduction in the number of female embryos, it might actually be the opposite: in WT, 19 out of 32 embryos are female (59%), while for the 245kb-INV allele 9 out of 13 embryos are female (69%). In our experience, to properly evaluate deviations to sex ratios in mouse litters, a much higher number of embryos (n>100) needs to be collected; for the scope of this study, we do not think such numbers are necessary. Nevertheless, in line with the reviewer's suggestion, we genotyped and analysed two additional litters (11 additional embryos) to evaluate how the trend would evolve. We now have 17 female embryos out of a total of 24, so 71%, suggesting that indeed sex ratio might be distorted for the 245kb-INV in favor of female embryos. These additional embryos were also analysed for *Atp7a* and *Xist* expression; these results were included in the manuscript (Fig. 4) and do not change the previous conclusions, except that now the increased *Xist* expression from the 245kb-INV is statistically significant. We have changed the text accordingly.

Finally, regarding the analysis of mutant female mESC being more informative than that of male mESC: we very much agree with the reviewer; however, our attempts to generate "clean" heterozygous lines for this inversion failed repeatedly. The clones we succeeded in generating (for female mESC) harboured the intended inversion on one chromosome; but on the other allele, we

© 2022. Published by The Company of Biologists under the terms of the Creative Commons Attribution License (https://creativecommons.org/licenses/by/4.0/).

detected indels and sometimes larger rearrangements associated with the sites targeted by the CRISPR/Cas9 system, which would compromise our analysis regarding *Xist* regulation. We never observed homozygous inversions. These results were also part of the reason we decided to generate the corresponding alleles in mice, with which a “clean” heterozygous genotype can be achieved (at least more easily).

3. This study found that the Tsix-TAD inversion leads to upregulated Xist expression in male ESCs and female F1 embryos. The authors suggest that the cause of Xist upregulation could be the result of altered orientation of the CTCF sites within the TAD inversion. However, the authors did not directly test this hypothesis. One suggestion would be to investigate which CTCF sites are responsible for Xist upregulation, by performing TAD inversions on smaller regions of the Tsix-TAD. Using a loss of function, and also perhaps gain of function approaches for specific CTCF sites within this Tsix-TAD would reveal a hierarchy of TAD strength within this region of the Xic, and may also result in larger gene expression changes.

Answer #16: We thank the reviewer for this suggestion. We agree that further dissecting specific CTCF sites within the Tsix-TAD, especially the ones within *Linx* and *Chic1*, could be informative regarding the structural organization and regulation of the *Xic*.

We have previously generated deletions including three CTCF sites within *Linx* in male mESC (Galupa et al., 2020). For reference, we reproduce the respective figure below. 5C analysis revealed (i) increased contacts between the *Linx* 3’end region and the *Chic1* locus (see B), which harbours CTCF sites in convergent orientation to those within the *Linx* 3’end region; and (ii) decreased contacts between the *Linx* 3’end region and *Xite*, as well as decreased basal contacts throughout the Xist-TAD (see C, and arrowhead). Despite these structural rearrangements, no effect was observed on *Xist* expression (see D).

Figure from (Galupa et al., 2020)

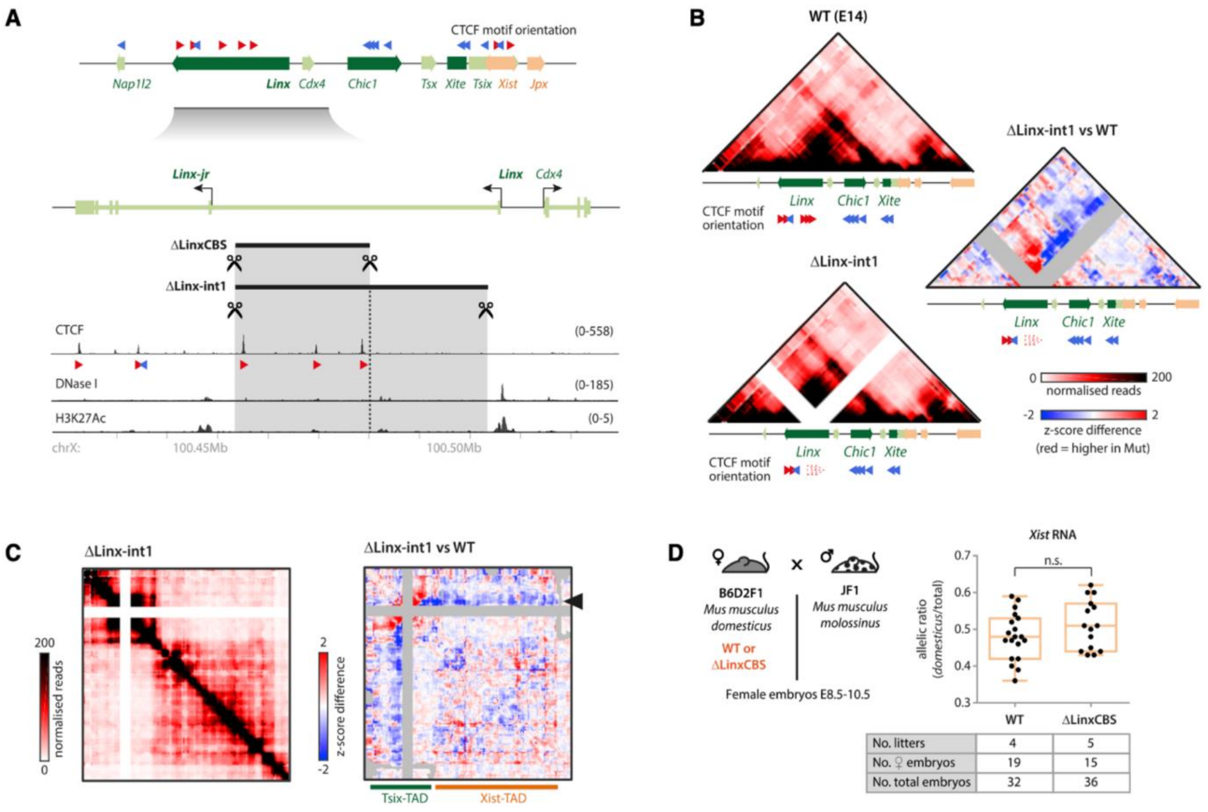

	WT	ΔLinxCBS
No. litters	4	5
No. ♀ embryos	19	15
No. total embryos	32	36

To address the reviewer’s comment, we generated and analysed an inversion of the same 3 CTCF sites that we previously deleted. We also set out to investigate the impact of deleting CTCF binding sites within the *Chic1* locus. These new results were included in the manuscript as a new section in the Results, and compiled in two new figures (Fig. 5 and Fig. 6). We can also not include these

© 2022. Published by The Company of Biologists under the terms of the Creative Commons Attribution License (https://creativecommons.org/licenses/by/4.0/).

results if the reviewer(s) find it more appropriate to do so. We reproduce the text and figures below:

Mutating clusters of CTCF sites within Linx *and* Chic1 *lead to changes in* Xist *expression*

To further explore the link between the topological organization of the Tsix-TAD and *Xist* regulation, we decided to generate alleles with deletions and/or inversions of the clusters of CTCF sites within *Linx* and within *Chic1*. We have previously deleted a large intronic interval containing three *Linx* CTCF sites, in male ESCs (~51 kb) and in mice (~25 kb) (Galupa et al., 2020), which led to some alterations in the topological organization of the two *Xic* TADs but no changes in *Xist* expression in female embryos. We decided to test the impact of inversions of exactly the same regions, in male mESCs (Linx-51kb-INV) and in mice (Linx-25kb-INV) (**Fig. 5A**). 5C analysis of the Linx-51kb-INV allele revealed higher frequency of contacts between the now inverted *Linx* locus and regions immediately upstream (**Fig. 5B,** black arrowhead), and lower frequency of contacts between (inverted) *Linx* and *Chic1*, *Xite* and elements within the Xist-TAD (**Fig. 5B-C,** blue arrowhead), in agreement with the change of orientation of the three *Linx* CTCF sites. These results are reminiscent of what we observed for the 245kb-INV allele (**Fig. 2B-C**), and they support the hypothesis that loss of contacts with the Xist-TAD in the 245kb-INV allele is associated to inversion of the CTCF sites within *Linx*. Consistently, analysis of insulation scores across

© 2022. Published by The Company of Biologists under the terms of the Creative Commons Attribution License (https://creativecommons.org/licenses/by/4.0/).

Figure 5

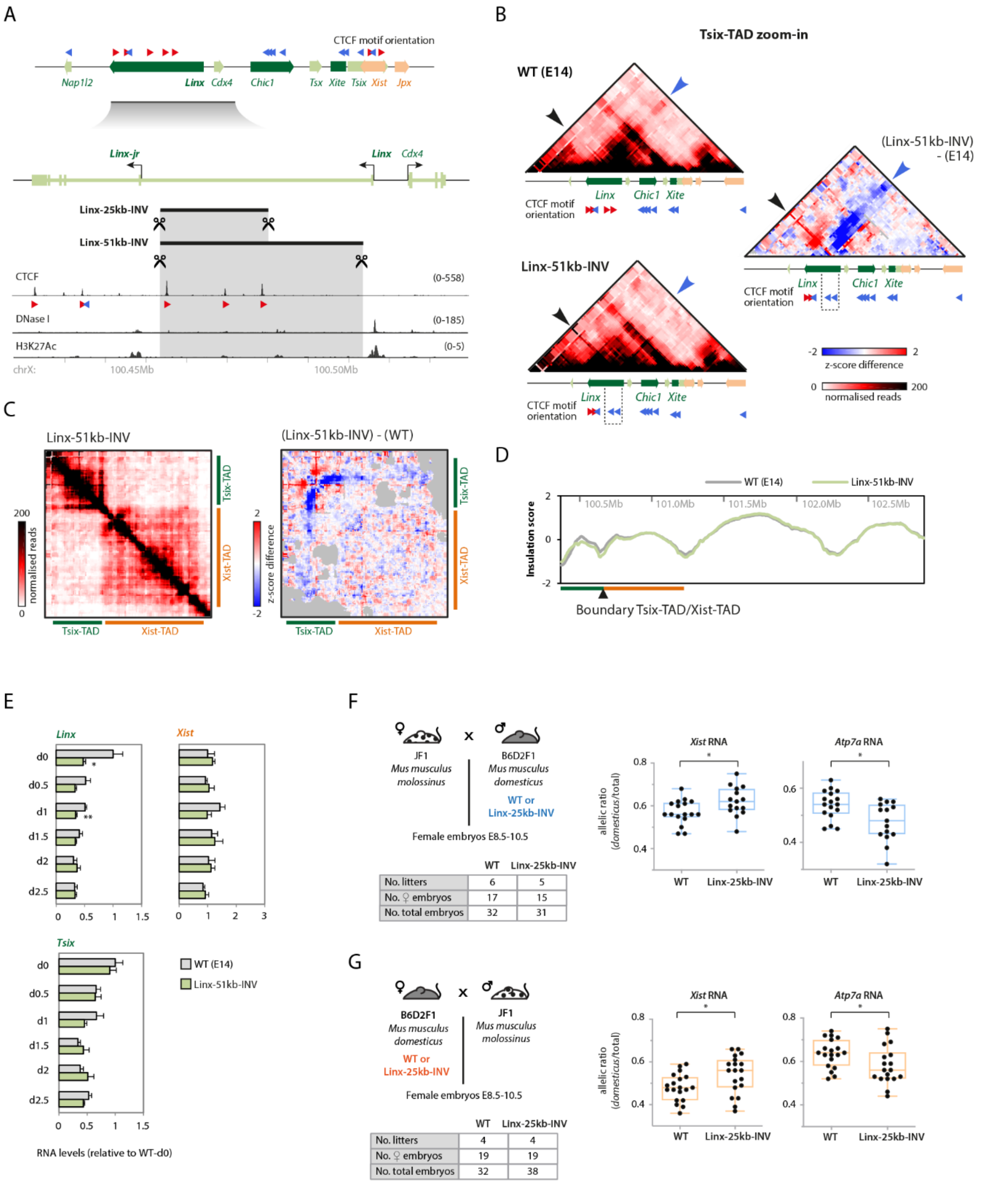

© 2022. Published by The Company of Biologists under the terms of the Creative Commons Attribution License (https://creativecommons.org/licenses/by/4.0/).

the TADs revealed a gain of insulation across the boundary between the Tsix-TAD and the Xist-TAD (**Fig. 5D**), though less pronounced than what we observed for 245kb-INV allele (**Fig. 2D**). We next analysed gene expression across the *Xic* for the Linx-51kb-INV mESC in the pluripotent state (d0) and during early differentiation (d0.5-d2.5); expression of *Linx* was significantly downregulated at some time points (**Fig. 5E**) but no changes were observed for *Xist* or *Tsix* (**Fig. 5E**) nor for any other locus across the *Xic* (data not shown). However, when we analysed *Xist* expression in female embryos carrying an heterozygous Linx-25kb-INV allele, we observed significantly higher expression of *Xist* for the inverted allele, for both paternal and maternal transmission (**Fig. 5F-G**), and also corresponding decrease in expression of the X-linked gene *Atp7a* (**Fig. 5F-G**), suggestive of skewed XCI compared to control. Overall, the inversion of the *Linx* CTCF cluster leads to similar phenotypes compared to the large 245kb inversion, namely a decrease in contact frequency between *Linx* and the Xist-TADs, and a concomitant gain of insulation between them, and increased *Xist* expression in cis in female embryos.

We have previously generated in male mESC a ~4kb deletion within *Chic1* (Giorgetti et al., 2014) that encompassed two of the three CTCF binding sites present in the locus (**Fig. 6A**), but we did not study its impact on chromosome conformation nor on *Xist* expression, which we set out to do here. Differential 5C analysis between this Chic1-4kbΔ allele and wildtype revealed showed a reduction in contacts between *Chic1* and *Linx*, and also between *Chic1* and *Xite* (**Fig. 6B**), consistent with loss of the *Chic1* CTCF sites. We also noted a seemingly increase in contact frequency between *Linx* and *Xite* (**Fig. 6B, Fig. S1**), which would be consistent with a model of competition between *Chic1* and *Xite* CTCF sites to form loops with the CTCF sites within *Linx*. However, these differences in contact frequencies overall remained rather close to the "noise" levels of the 5C map. We wondered whether these effects would be more pronounced if the remaining CTCF binding site was also removed; thus, we generated, in male mESC, a larger deletion (Chic1-14kbΔ) encompassing all three CTCF sites within Chic1 (**Fig. 6A**). We observed similar contact rearrangements within the Tsix-TAD as for Chic1-4kbΔ but more pronounced (**Fig. 6C**), suggesting that it is the loss of the CTCF sites that underlies the observed topological differences. To study the impact of these deletions on gene expression across the *Xic*, we profiled transcript levels as done previously in the pluripotent state (d0) and during early differentiation. Expression of *Chic1* itself was consistently upregulated in both Chic1-4kbΔ and Chic1-14kbΔ (**Fig. 6D- E**); it is intriguing to think that this could potentially be linked to its now shorter length, as shorter genes have been associated to higher levels of expression (Castillo-Davis et al., 2002; Chiaromonte et al., 2003). We also observed higher expression of the gene upstream of *Chic1*, *Cdx4*: interestingly, the effects seemed to scale up with the larger deletion – in Chic1-4kbΔ mESC, there was a slight increase in *Cdx4* levels across time points but not statistically significant, while in Chic1-14kbΔ the increase was more pronounced and statistically significant for some of the time points. This effect could be connected to the removal of all CTCF sites from within the *Chic1* locus, which could potentially "shield", or insulate, *Cdx4* from activating influences downstream of the CTCF sites. Expression of *Xist* expression was also more affected in mESC containing the larger deletion: we observed a mostly consistent downregulation across all time points, but this effect was not statistically significant in this context. In female embryos, however, we did observe a statistically significant decrease in *Xist* expression from the deletion alleles (**Fig. 6D-E**), and more pronounced for the Chic1-14kbΔ allele. This suggests that the *Chic1* CTCF cluster might operate to favour *Xist* expression in cis. These results potentially illustrate as well how loss of one additional CTCF binding site might be enough to cause stronger changes in chromosome conformation and gene expression.

Together, our results on inverting or deleting *Linx* and *Chic1* CTCF clusters highlight the rather complex regulatory landscape within the *Xic*. Similar to the 245kb inversion, these mutant alleles reveal how *Xist* is sensitive to changes involving CTCF binding sites within the neighbouring Tsix-TAD. These results also suggest that the phenotypes observed in the 245kb-INV mESC are likely a compound of effects from changing different elements within the Tsix-TAD.

© 2022. Published by The Company of Biologists under the terms of the Creative Commons Attribution License (https://creativecommons.org/licenses/by/4.0/).

Figure 6

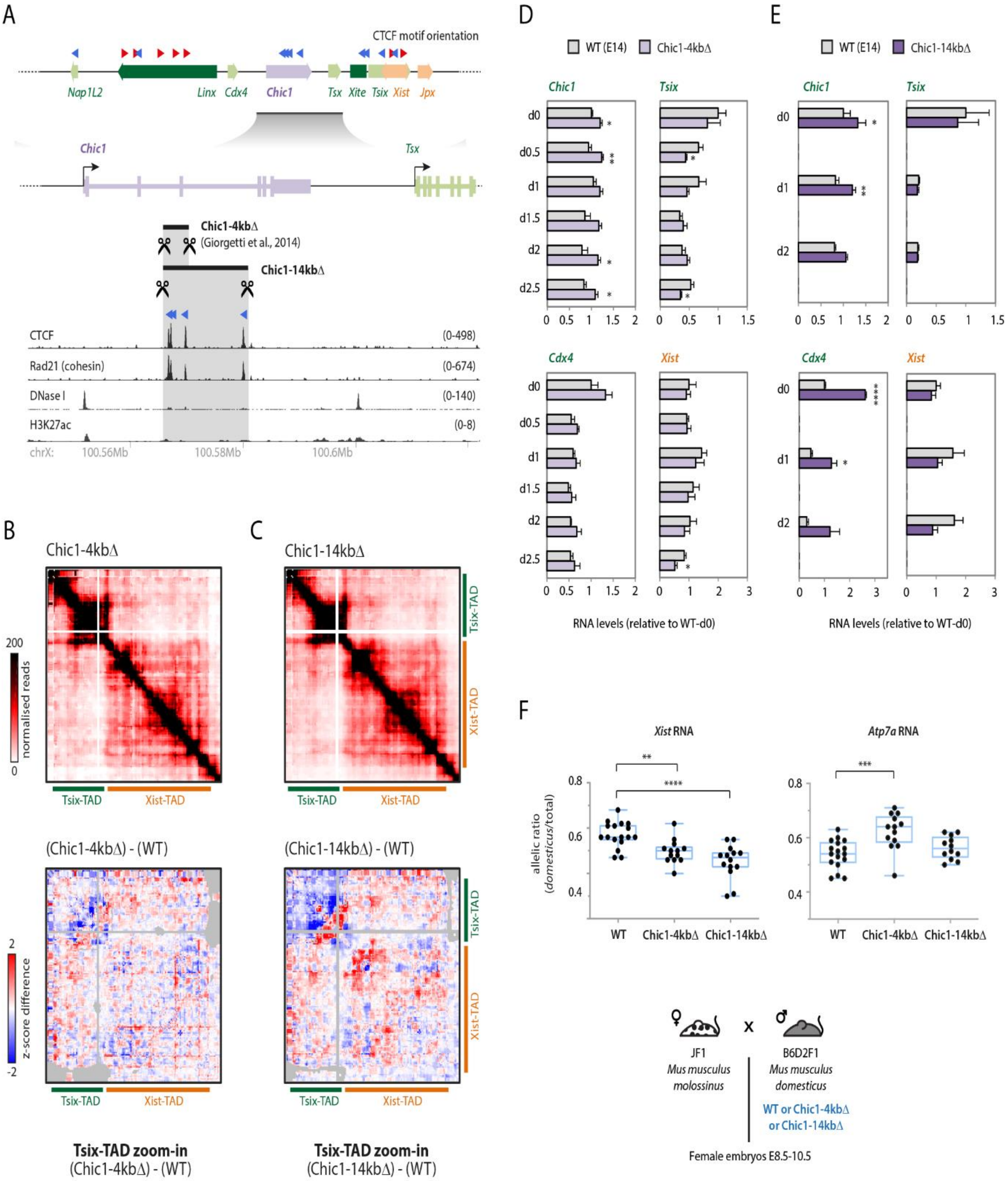

	WT	Chic1-4kbΔ	Chic1-14kbΔ
No. litters	6	4	5
No. ♀ embryos	17	13	13
No. total embryos	32	24	25

© 2022. Published by The Company of Biologists under the terms of the Creative Commons Attribution License (https://creativecommons.org/licenses/by/4.0/).

Original submission

First decision letter

MS ID#: DEVELOP/2022/200568

MS TITLE: Inversion of a topological domain leads to restricted changes in its gene expression and affects inter-domain communication

AUTHORS: Rafael Galupa, Christel Picard, Nicolas Servant, Elphege P Nora, Yinxiu Zhan, Joke van Bemmel, Fatima El Marjou, Colin Johanneau, Maud Borensztein, Katia Ancelin, Luca Giorgetti, and Edith Heard
ARTICLE TYPE: Research Article

Thank you for sending your manuscript to Development through Review Commons.

I am happy to tell you that your manuscript has been accepted for publication in Development, pending our standard ethics checks.

Reviewer 1

Advance summary and potential significance to field

This study investigates phenotypes resulting from a TAD inversion at the X-inactivation center (Xic). The authors generated a 245kb inversion of the Tsix-TAD, which includes various CTCF clusters, and excludes the Xite and Tsix genes.
Importantly, the inversion maintained the boundaries intact. The authors introduced this inversion into male embryonic stem cells (ESCs) and also made a transgenic mouse with this construct. They discovered that the inversion affected the contact insulation with the neighboring TAD. The inversion did not affect expression of most genes, except for Xist, which was upregulated in this inversion mutant. The strength of this manuscript is the clear demonstration of TAD-TAD interactions that can impact gene expression important for gene development.

Comments for the author

This manuscript is well-written, and has some exciting data that will advance the field of nuclear organization and X-chromosome Inactivation.
The authors have made edits from the previous version that I reviewed, and I believe these edits further support and strengthen their conclusions. I have no further edits or comments to make, and I support publication of this manuscript.

Reviewer 2

Advance summary and potential significance to field

Galupa et al. use a large inversion inside the Tsix TAD to investigate the role of chromatin contact rearrangements within TADs on the regulation of this locus. The results are largely consistent with current models of gene regulation by distal regulatory elements, and are generally well supported by the figures and data shown.

Comments for the author

The authors have satisfactorily addressed all my questions and I do not have any additional concerns about the work.

© 2022. Published by The Company of Biologists under the terms of the Creative Commons Attribution License (https://creativecommons.org/licenses/by/4.0/).
